# Equilibrium Control in Uncertain Linear Quadratic Differential Games with V-Jumps and State Delays: A Case Study on Carbon Emission Reduction

**DOI:** 10.3390/e26110943

**Published:** 2024-11-04

**Authors:** Zhifu Jia

**Affiliations:** School of Mathematics and Sciences, Suqian University, Suqian 223800, China; jzflzbx@nuaa.edu.cn

**Keywords:** differential game optimistic value problems, Nash equilibrium, saddle point equilibrium

## Abstract

Uncertainty, time delays, and jumps often coexist in dynamic game problems due to the complexity of the environment. To address such issues, we can utilize uncertain delay differential equations with jumps to depict the dynamic changes in differential game problems that involve uncertain noise, delays, and jumps. In this paper, we first examine a linear quadratic differential game optimistic value problem within an uncertain environment characterized by jumps and delays. By applying the Z(x,y) transform, we convert the infinite-dimensional problem into a finite-dimensional one. We then demonstrate that the condition for the existence of a Nash equilibrium strategy is equivalent to the existence of solutions to two cross-coupled matrix Riccati equations. Furthermore, we explore the saddle point equilibrium strategy of the linear quadratic differential game optimistic value model and derive the corresponding saddle point equilibrium solution. Finally, we apply our results to solve a carbon emission reduction game problem.

## 1. Introduction

The uncertainty of experts’ experience, particularly in relation to belief degrees [1] as expressed by experts, is a significant factor when dealing with phenomena that are indeterminate. This uncertainty can have profound implications on decision making and modeling processes, especially when the phenomena in question do not have clear, definitive outcomes. In addressing such indeterminate scenarios, incorporating a measure of this uncertainty becomes essential for more accurate analysis and predictions. To effectively capture the dynamics of phenomena that are uncertain in nature, particularly with respect to varying belief degrees, Liu [2] introduced a novel mathematical framework known as the uncertain differential equation, which is grounded in what is now called the Liu process. This process provided a structured way to model the uncertainty inherent in these phenomena. Subsequent research [3] expanded upon Liu’s initial proposal, exploring and deepening the understanding of the properties and behaviors of the Liu process, and allowing for more robust applications by uncertain differential systems driven by it. Uncertain differential equations (UDEs) and stochastic differential equations (SDEs) both deal with differential systems that exhibit some degree of randomness or uncertainty. However, they differ in their mathematical formulation and interpretation. SDEs utilize probability theory and Brownian motion to model randomness, effectively capturing the intrinsic variability and unpredictability inherent in certain phenomena. In contrast, UDEs utilize uncertainty theory, which is based on a non-additivity measure called belief degree, to describe systems with inherent indeterminacy that cannot be fully characterized by probabilistic methods. This distinction makes UDEs particularly suitable for situations where traditional probabilistic models may be inadequate or inappropriate.

In later studies, uncertain delay differential equations emerged as a significant area of interest. These equations, which incorporate time delays into uncertain systems, offered a more nuanced approach to modeling scenarios where the effects of a current state are delayed and manifest over time. This complexity attracted attention for its realism in capturing delayed responses in uncertain environments. In uncertain delay differential equations, “delay” signifies that the rate of change of the dependent variable at any time depends not only on its current value but also on a prior state. The delay represents processes where present dynamics are influenced by past states, a crucial feature for modeling temporal dependencies in control systems. Research on uncertain delay differential equations concentrated on four main aspects: solution existence, solution uniqueness, system stability under uncertainty and delays, and potential applications. Understanding these characteristics was vital to establishing whether solutions were reliable, how they evolved, and how systems could be controlled to achieve desired outcomes in the presence of uncertainty and delays. Addressing these phenomena, Barbacioru [4] utilized uncertainty functional differential equations to model volatility in financial markets, particularly for dynamic systems influenced by past states. Ge et al. [5] established existence and uniqueness theorems for uncertain delay differential equations under specific conditions, forming a theoretical foundation that ensured meaningful and interpretable solutions. Building on prior work, Wang et al. [6,7] introduced concepts such as stability in measure, mean, and p-th moment to describe the stability of uncertain delay differential equations, while Jia et al. [8] focused on stability in distribution. In this context, stability indicates whether the system remains predictable and well behaved over time, even with uncertainty. Their contributions included stability theorems that clarified conditions for system integrity despite fluctuating degrees of uncertainty. Chen, Zhu [9], and Jiang [10] investigated optimal control in uncertain systems with time delays, excluding sudden jumps. They employed expected value criteria, a common method where decisions are based on the weighted average of possible outcomes. Their research sought optimal control strategies to minimize risks and maximize desired outcomes, even in the face of uncertainty and delays.

It is widely recognized that uncertain processes can be subject to sudden, unpredictable changes brought on by emergencies or crises. These events can include economic downturns, pandemics, natural disasters like earthquakes, or geopolitical events such as wars. Such unexpected occurrences can drastically alter the behavior of a system, introducing a new layer of complexity and requiring models that can accommodate these sudden shifts. In response to the need for models that could account for abrupt changes in uncertain systems, Deng et al. [11] introduced the concept of *V*-jumps. These jumps represent significant, discrete changes in the state of the system that occur unpredictably. The *V*-jumps framework has since been widely adopted as an effective tool for describing the evolution of phenomena that experience sudden shifts in response to emergencies or other unforeseen events. Furthering the theoretical foundation of *V*-jumps, Deng et al. [12] provided mathematical proof of the existence and uniqueness of solutions to uncertain differential equations that incorporate *V*-jumps. This result was crucial because it ensured that the models used to describe systems with jumps could produce reliable and consistent outcomes, even when dealing with abrupt changes in system behavior. Following the development of uncertain differential equations with *V*-jumps, these mathematical models became widely adopted in the field of optimal control, particularly in situations where sudden, unpredictable changes, or jumps, occur within a system. The application of *V*-jumps allowed for more realistic and nuanced modeling of systems that are prone to abrupt shifts in state, making them especially useful in optimizing control strategies under uncertain conditions. One notable example of this application is found in the work of Deng [13,14,15], who explored uncertain optimal control models incorporating jumps. Deng also extended this investigation to linear quadratic models with jumps, all under the expected value evaluation criteria. These models were designed to minimize the cost or maximize the efficiency of control in systems where both linear dynamics and sudden jumps played significant roles, with the expected value approach providing a means of balancing the trade offs between different outcomes. Further extending the reach of uncertain jump models, Chen et al. [16] studied the optimal control of uncertain uncertain dynamic systems with jumps, applying their findings to practical scenarios such as advertising models. This work demonstrated the usefulness of such models in optimizing strategies for dynamic, unpredictable environments like the advertising market, where shifts in consumer behavior or market conditions can cause sudden changes in outcomes. In general terms, the expected value model is an effective measurement tool when assessing uncertain systems. By using the concept of a weighted average, this model provides a pragmatic way to account for various possible outcomes, assigning each one a belief degree and calculating the expected or average result. This approach works particularly well in situations where the range of possible outcomes is not extreme, and where a balanced, middle-ground prediction is appropriate for decision making. However, in cases where data polarization, meaning the outcomes are highly skewed or concentrated around extreme values, becomes a significant factor, or when other unique characteristics of uncertain variables must be considered, the optimistic value model offers a more suitable alternative. Unlike the expected value model, which seeks a middle ground, the optimistic value model focuses on the most favorable outcomes, making it more appropriate when decision makers prioritize the best case scenarios in uncertain environments. In contrast to the expected value approach, Deng and Chen [17,18], along with Deng et al. [19], conducted extensive research into optimistic value models within the context of uncertain optimal control with jumps. Their work aimed to explore how optimal control strategies could be devised when the focus shifts from an average, balanced outcome to a more optimistic outlook, prioritizing the potential for the best possible results despite the presence of uncertainties and sudden jumps in the system.

Building on the foundation of uncertain optimal control, Sun et al. [20] and Li et al. [21] expanded their research to address differential game problems under uncertainty, focusing specifically on the optimistic value criterion. Their study examined a counter-terror economic game model in an uncertain setting, though without the added complexity of jumps or delays. This work aimed to apply the optimistic value criterion in game theory, enabling strategic decision making in scenarios dominated by uncertainty. Jia et al. [22,23] subsequently broadened the scope by investigating uncertain differential games involving multiple factors and, notably, uncertain jumps in a multidimensional context. Their research offered significant insights into modeling and control of strategic interactions where multiple dimensions and abrupt changes introduce further unpredictability. In the realm of uncertain stochastic jump systems, Jia and Liu [24] explored a hybrid differential game system with V-n jumps, a form of sudden shift in the system. They introduced equilibrium strategies such as saddle point equilibria, indicating states where neither player can unilaterally gain an advantage. This theoretical advancement was applied to a practical case of an advertising duopoly game, where two firms compete in a market marked by stochastic elements and sudden shifts. Chen et al. [25] further advanced the field by analyzing optimal control and zero-sum games in systems characterized by multifactor uncertainty, where each factor could experience sudden jumps. Their research tackled these challenges, presenting strategies to optimize outcomes in highly unpredictable conditions. Besides their work on differential games, Jia et al. [26] contributed to the mathematical foundation by proving theorems on the existence and uniqueness of solutions for uncertain delay differential equations with jumps, ensuring that these models are mathematically robust under specified conditions. Real-world differential game problems are often characterized by combinations of uncertainty, time delays, and abrupt events. This complex setting requires adaptive models that account for uncertain, delayed, and sudden occurrences. To address these complex game problems, we will employ uncertain delay differential equations with jumps. These equations capture the dynamics of differential games influenced by Liu noises, time delays, and abrupt changes, all under the optimistic value criterion. This approach enables a focus on favorable outcomes in a highly uncertain environment, allowing for more strategic and optimistic decision making.

The contributions of this paper are: (1) to obtain the Nash equilibrium of an uncertain delay linear quadratic differential game optimistic value model with a jump; (2) to obtain the saddle equilibrium of an uncertain delay linear quadratic differential game optimistic value model with a jump. According to different optimization purposes, the expected value and optimistic value are different evaluation criterion. The results [9] of the expected model for uncertain control systems cannot be directly used for an optimistic value model. Compared with the models in Ref. [9], we extend the results to the uncertain delay linear quadratic differential game optimistic value model with a jump. Compared with the models in Ref. [20], we introduce jump factors and delays, and extend the model to the multidimensional case. In addition, we also consider Nash equilibrium solutions, and the optimistic value models in our paper are more complex and more practical due to the existences of jumps and delays; and (3) to apply the model to solve a carbon emission reduction game problem.

The structure of this paper is organized as follows. Section 2 will collect some preliminary results including the β-optimistic value, Liu process, V-jump process, and so on, which are essential for our analysis. In Section 3, we pose a linear quadratic differential game optimistic value problem under an uncertain jump and delay environment. By Z(x,y) transform, the infinite dimension problem is transformed into a finite dimension; then, we obtain the existence condition of the Nash equilibrium strategy that is equivalent to the existence solution of two cross-coupled matrix Riccati equations. Section 4 is devoted to the study of the saddle point equilibrium problem of the uncertain delay linear quadratic differential game optimistic value model with a jump. The application is given to show the validity of the obtained results in Section 5.

## 2. Preliminaries

In this section, we review some preliminaries including the β-optimistic value, Liu process, V-jump process, and so on.

**Definition 1**  ([4])**.** *Let ξ be an uncertain variable, M be an uncertain measure, and the confidence level β∈(0,1]. Then, $\xi_{\sup}\left(\beta \right)=\sup\left\{r|M\left\{\xi \geq r\right\}\geq \beta \right\}$ is called the β-optimistic value to ξ; and $\xi_{\inf}\left(\beta \right)=\inf\left\{r|M\left\{\xi \leq r\right\}\geq \beta \right\}$ is called the β-pessimistic value to ξ.*

**Definition 2**  ([4])**.** *An uncertain process $C_{t}$ is a Liu process with respect to time t if it satisfies the following:*

*(a)*  *$C_{0}=0$ and almost all sample paths are Lipschitz continuous;*
*(b)*  *$C_{t}$ is in independent increments and stationary;*
*(c)*  *Every increment $C_{s+t}-C_{s}$ is a normal uncertain variable with expected value 0 and variance $t^{2}$, and its uncertainty distribution is*
  $$\Phi \left(x\right)={\left(1+\exp\left(\frac{-\pi x}{\sqrt{3}t}\right)\right)}^{-1},\;\;x\in R.$$

**Remark 1.**  *Liu [27] challenged the widely accepted presumption of Ito’s calculus in 2013, and believed that Liu’s calculus is a potential tool for the stock market. For example, Liu et al. [28] considered the real Alibaba stock prices from 1 January 2019 to 30 June 2020. Ye et al. [29] considered USD–CNY exchange rates from 1 October 2019 to 30 June 2021. The experiments showed that the residuals are far from frequency stability, and should not be regarded as random variables, but rather as uncertain variables. At the same time, the uncertain differential equations driven by the Liu process were very suitable for simulating these two real cases.*

The definition of *V*-jump uncertain process is as follow:

**Definition 3**  ([11])**.** *A V-jump uncertain process with parameters $\vartheta_{1}$ and $\vartheta_{2}$ $\left(0<\vartheta_{1}<\vartheta_{2}<1\right)$ for t≥0 is defined if*

*(i)*  $V_{0}=0;$
*(ii)*  *$V_{t}$ has stationary and independent increments;*
*(iii)*  *For any given time t>0, every increment $V_{s+t}-V_{s}$ is a Z jump uncertain variable $\xi ∼Z(\vartheta_{1},\vartheta_{2},t)$ for ∀s>0, whose uncertainty distribution is*
  $$\begin{array}{c} \Phi \left(x\right)=\left\{\begin{array}{cc} 0 & if\;x<0,\\ \frac{2\vartheta_{1}}{t}x & if\;0\leq x<\frac{t}{2},\\ \vartheta_{2}+\frac{2(1-\vartheta_{2})}{t}\left(x-\frac{t}{2}\right) & if\;\frac{t}{2}\leq x<t,\\ 1 & if\;x\geq t. \end{array}\right. \end{array}$$

**Definition 4**  ([12])**.** *Suppose that $C_{t}$ is an uncertain Liu process with respect to time t, $V_{t}$ is an uncertain V-jump process with respect to time t, and ρ,ς, and ν are some given functions about $X_{t}$ and t. Then,*

$$dX_{t}=\rho \left(t,X_{t}\right)dt+\varsigma \left(t,X_{t}\right)dC_{t}+\nu \left(t,X_{t}\right)dV_{t}$$

*is called an uncertain differential equation with a V-jump.*

Deng et al. [19] presented the following general multidimensional uncertain optimal control model with V-n jumps:

(1) $$\begin{array}{c} \left\{\begin{array}{c} J\left(t,x\right)=\underset{\omega_{s}\in \Omega }{\sup}{\left\{\int_{t}^{T}F\left(s,X_{s},\omega_{s}\right)ds+h\left(T,X_{T}\right)\right\}}_{\sup}\left(\beta \right)\\ \;s.\;\;t.\;\\ \;dX_{s}=\rho \left(s,X_{s},\omega_{s}\right)ds+\;\varsigma \left(s,X_{s},\omega_{s}\right)dC_{s}+\;\nu \left(s,X_{s},\omega_{s}\right)dV_{s}\\ \;X_{t}=x, \end{array}\right. \end{array}$$

where $X_{s}=\left(X_{s1},X_{s2},\cdots ,X_{sn}\right)$ is an n-dimensional state vector with the initial condition $X_{t}=x=\left(x_{1},x_{2},\cdots ,x_{n}\right)$ at time *t*, $\omega_{s}$ is the decision vector of dimension *m* (represents the control function $\omega (s,X_{s})$ of time *s* and state $X_{s}$) in a domain Ω, $F:\left[0,T\right)\times R^{n}\times R^{m}\to R$ the objective function, and $h:\left[0,T\right)\times R^{n}\to R$ is the terminal reward function. In addition, $\rho :\left[0,T\right)\times R^{n}\times R^{m}\to R$ is a column-vector function about *s*, $X_{s}$, and $\omega_{s}$, $\varsigma :\left[0,T\right)\times R^{n}\times R^{m}\to R^{n}\times R^{l}$ and $\nu :\left[0,T\right)\times R^{n}\times R^{m}\to R^{n}\times R^{l}$ are two matrix functions about *s*, $X_{s}$, and $\omega_{s}$, and $C_{s}=\left(C_{s1},C_{s2},\cdots ,C_{sl}\right)$, $V_{s}=\left(V_{s1},V_{s2},\cdots ,V_{sl}\right)$, where $C_{s1},C_{s2},\cdots ,C_{sl}$ are independent Liu processes, $V_{s1},V_{s2},\cdots ,V_{sl}$ are independent V-n jumps processes, and $C_{si}$ and $V_{sj}$ for any i,j=1,2,⋯,l(i≠j) are independent, and the final time T>0 is fixed or free.

**Definition 5**  ([19])**.** *Suppose $V_{t}$ is a V-jump process with parameters $\vartheta_{1}$ and $\vartheta_{2}$, and let $\eta =V_{t}=V_{t+\Delta t}-V_{t}$. Then, the β-optimistic value and β-pessimistic value are defined for any belief degree β∈(0,1)*

$$\begin{array}{c} \eta_{\sup}\left(\beta \right)=\left\{\begin{array}{cc} (1-\frac{\beta }{2(1-\vartheta_{2})})\Delta t, & if\;0<\beta <1-\vartheta_{2}\\ \frac{\Delta t}{2}, & if\;1-\vartheta_{2}\leq \beta <1-\vartheta_{1}\\ \frac{1-\beta }{2\vartheta_{1}}\Delta t, & if\;1-\vartheta_{1}\leq \beta <1, \end{array}\right. \end{array}$$

*and*

$$\begin{array}{c} \eta_{\inf}\left(\beta \right)=\left\{\begin{array}{cc} \frac{\beta }{2\vartheta_{1}}\Delta t, & if\;0<\beta \leq \vartheta_{1}\\ \frac{\Delta t}{2}, & if\;\vartheta_{1}<\beta \leq \vartheta_{2}\\ (1-\frac{1-\beta }{2(1-\vartheta_{2})})\Delta t, & if\;\vartheta_{2}<\beta <1, \end{array}\right. \end{array}$$

*respectively.*

**Lemma 1**  ([19])**.** *If J(t,x) exists and is twice differentiable on $\left[0,T\right]\times R^{n}.$ Then, we have*

(2) $$\begin{array}{c} -J_{t}=\underset{\omega_{t}}{\sup}\left\{F+\nabla_{x}J^{\tau }\rho +\frac{\sqrt{3}}{\pi }\ln\frac{1-\beta }{\beta }∥\nabla_{x}J^{\tau }{\varsigma ∥}_{1}+k∥\nabla_{x}J^{\tau }\nu ∥\right\}, \end{array}$$

*where $J_{t}\left(t,x\right)$ is the partial derivatives of the function J(t,x) in t. $\nabla_{x}J\left(t,x\right)$ is the gradient of J(t,x) in x,τ represents the transpose, and ${∥\cdot ∥}_{1}$ and ∥·∥ are the 1-norm for vectors, that is, ${∥\cdot ∥}_{1}=\sum_{i=1}^{n}\left|\kappa_{i}\right|$ and $∥\cdot ∥=\sum_{i=1}^{n}\kappa_{i}$ for $\kappa =(\kappa_{1},\kappa_{2},\cdots ,\kappa_{k}),$ respectively. ρ,ς,ν are defined the same as Equation (1), and $F,\;\rho ,\;\varsigma ,\;\nu ,\;J_{t},\nabla_{x}J$ denote $F(t,x,\omega_{t}),$ $\rho (t,x,\omega_{t}),$ $\varsigma (t,x,\omega_{t}),$ $\nu (t,x,\omega_{t}),$ $J_{t}\left(t,x\right),$ $\nabla_{x}J\left(t,x\right),$ respectively, and*

*(1)*  *if $\nabla_{x}J^{\tau }\nu \geq 0$ (all terms are nonnegative), then $k=1-\frac{\beta }{2(1-\vartheta_{2})}\left(0<\beta <1-\vartheta_{2}\right)$ or $k=\frac{1}{2}\left(1-\vartheta_{2}\leq \beta <1-\vartheta_{1}\right)$ or $k=\frac{1-\beta }{2\vartheta_{1}}\left(1-\vartheta_{1}\leq \beta <1\right)$;*
*(2)*  *if $\nabla_{x}J^{\tau }\nu <0$ (all terms are negative), then $k=\frac{\beta }{2\vartheta_{1}}\left(0<\beta \leq \vartheta_{1}\right)$ or $k=\frac{1}{2}\left(\vartheta_{1}<\beta \leq \vartheta_{2}\right)$ or $k=1-\frac{1-\beta }{2(1-\vartheta_{2})}\left(\vartheta_{2}<\beta <1\right)$.*

**Lemma 2.**  *Suppose $C_{t}$ is a Liu process and $V_{t}$ is a V-jump process, and let ψ(t,c,v) be a continuously differentiable function. Then, $\Upsilon_{t}=\psi \left(t,C_{t},V_{t}\right)$ is an uncertain process and*

$$\begin{array}{c} d\Upsilon_{t}=\frac{\partial \psi }{t}\left(t,C_{t},V_{t}\right)dt+\frac{\partial \psi }{c}\left(t,C_{t},V_{t}\right)dC_{t}+\frac{\partial \psi }{v}\left(t,C_{t},V_{t}\right)dV_{t}. \end{array}$$

Similar to the proof of Theorem 4 of Ref. [3], Lemma 2 can be easily obtained.

## 3. Main Results

Real-world differential game problems are often characterized by combinations of uncertainty, time delays, and abrupt events. This complex setting requires adaptive models that account for uncertain, delayed, and sudden occurrences. To address these complex game problems, we consider the following linear uncertain delay differential systems with V-n jumps. We separately outline the system’s dynamics, constraints, and objectives for each player in the Nash equilibrium framework.

(3) $$\begin{array}{c} \;\left\{\begin{array}{c} \;dX_{s}=\left\{A_{1}\left(s\right)X_{s}+A_{2}\left(s\right)Y_{s}+A_{3}\varrho_{s}+B_{1}\left(s\right)\omega_{1}\left(s\right)+B_{2}\left(s\right)\omega_{2}\left(s\right)\right\}ds+\{A_{4}\left(s\right)X_{s}\\ \;\;\;\;+A_{5}\left(s\right)Y_{s}\}dC_{s}+\left\{A_{6}\left(s\right)X_{s}+A_{7}\left(s\right)Y_{s}\right\}dV_{s},\;s\in \left[t,T\right]\\ \;Y_{s}=\int_{-d}^{0}e^{\lambda u}X_{s+u}du,\;\varrho_{s}=X_{s-d},\;s\in \left[t,T\right]\\ \;X_{s}=\varphi_{t}\left(s-t\right),\;t-d\leq s\leq t, \end{array}\right. \end{array}$$

where the matrices $A_{1}\left(s\right),A_{2}\left(s\right),A_{3},B_{1}\left(s\right),B_{1}\left(s\right),A_{4}\left(s\right),A_{5}\left(s\right),A_{6}\left(s\right)$, and $A_{7}\left(s\right)$ are appropriate size matrix functions, state $X_{s}$ (s≥−d) $\in R^{n}$ that started at time −d<0, *d* represents the delay, and λ is a constant. $C_{t}={\left(C_{t1},C_{t2},\cdots ,C_{tl}\right)}^{\tau }$ where $C_{t1},C_{t2},\cdots ,C_{tl}$ are independent Liu processes. $V_{t}={\left(V_{t1},V_{t2},\cdots ,V_{tl}\right)}^{\tau }$ where $V_{t1},V_{t2},\cdots ,V_{tl}$ are independent V-jump processes. The feasible strategies of the two players are: $\omega_{1}\left(s\right)\in \Omega_{1}$ and $\omega_{2}\left(s\right)\in \Omega_{2}$; $\Omega_{1}$ is the feasible strategy set of player 1 and $\Omega_{2}$ is the feasible strategy set of player 2. For a given confidence level β∈(0,1), the two players seek their respective optimal feasible strategies to maximize the following cost functions under the constraint of the state Equation (3), namely

(4) $$\begin{array}{cc} J_{1}\left(t,\varphi_{t}\right)= & \underset{\omega_{1}\left(s\right)\in \Omega_{1}}{\sup}\{\int_{t}^{T}[{\left(X_{s}+e^{\lambda d}A_{3}Y_{s}\right)}^{\tau }D_{1}\left(s\right)\left(X_{s}+e^{\lambda d}A_{3}Y_{s}\right)+\omega_{1}^{\tau }\left(s\right)R_{11}\omega_{1}\left(s\right)\\ \; & +\omega_{2}^{\tau }\left(s\right)R_{12}\omega_{2}\left(s\right){]ds+{\left(X_{T}+e^{\lambda d}A_{3}Y_{T}\right)}^{\tau }G_{1}\left(X_{T}+e^{\lambda d}A_{3}Y_{T}\right)\}}_{\sup}\left(\beta \right), \end{array}$$

(5) $$\begin{array}{cc} J_{2}\left(t,\varphi_{t}\right)= & \underset{\omega_{2}\left(s\right)\in \Omega_{2}}{\sup}\{\int_{t}^{T}[{\left(X_{s}+e^{\lambda d}A_{3}Y_{s}\right)}^{\tau }D_{2}\left(s\right)\left(X_{s}+e^{\lambda d}A_{3}Y_{s}\right)+\omega_{1}^{\tau }\left(s\right)R_{21}\omega_{1}\left(s\right)\\ \; & +\omega_{2}^{\tau }\left(s\right)R_{22}\omega_{2}\left(s\right){]ds+{\left(X_{T}+e^{\lambda d}A_{3}Y_{T}\right)}^{\tau }G_{2}\left(X_{T}+e^{\lambda d}A_{3}Y_{T}\right)\}}_{\sup}\left(\beta \right), \end{array}$$

where $D_{1},R_{11},R_{12},G_{1},D_{2},R_{21},R_{22},G_{2}$ are appropriate size matrix functions, $D_{1},D_{2}$ are the symmetric nonnegative definite matrix, and $R_{11},R_{12},R_{21},R_{22}$ are the symmetric positive definite matrix. Actually, the cost functions change with the initial time *t* and the initial state $\varphi_{t}$ change. Thus, we denote

$${\bar{J}}_{1}\left(\omega_{1}\left(s\right),\omega_{2}\left(s\right)\right)=J_{1}\left(t,\varphi_{t}\right),$$

$${\bar{J}}_{2}\left(\omega_{1}\left(s\right),\omega_{2}\left(s\right)\right)=J_{2}\left(t,\varphi_{t}\right),$$

where $\varphi_{t}\in C_{R}\left[-d,0\right]$ $\left(t=0,\varphi_{0}\in C_{R}\left[-d,0\right]\right)$ are the given initial functions and $C_{R}\left[-d,0\right]$ represents the space of all continuous functions on [−d,0] taking values in *R*.

The so-called Nash equilibrium problem is to find the feasible control strategies $\omega_{1}^{*}\left(t\right)$ and $\omega_{2}^{*}\left(t\right)$ under the system state (3)–(5) constraints such that

(6) $$\begin{array}{c} {\bar{J}}_{1}\left(\omega_{1}^{*}\left(t\right),\omega_{2}^{*}\left(t\right)\right)\geq {\bar{J}}_{1}\left(\omega_{1}\left(t\right),\omega_{2}^{*}\left(t\right)\right),\forall \omega_{1}\left(t\right)\in \Omega_{1}, \end{array}$$

(7) $$\begin{array}{c} {\bar{J}}_{2}\left(\omega_{1}^{*}\left(t\right),\omega_{2}^{*}\left(t\right)\right)\geq {\bar{J}}_{2}\left(\omega_{1}^{*}\left(t\right),\omega_{2}\left(t\right)\right),\forall \omega_{2}\left(t\right)\in \Omega_{2}. \end{array}$$

We note that the value functions $J_{1}\left(t,\varphi_{t}\right)$ and $J_{2}\left(t,\varphi_{t}\right)$ in (4) and (5) were defined in an infinite-dimensional space, so it is very difficult to solve it directly. We will transform the problem into a finite-dimensional space by constructing a new state. For this purpose, we use the same method as Ref. [9]. For Questions (3)–(7), we denote that

(8) $$\begin{array}{c} \left\{\begin{array}{c} \rho_{1}\left(t,x,y,\omega_{1},\omega_{2}\right)=A_{1}x+A_{2}y+B_{1}\omega_{1}+B_{2}\omega_{2},\\ \rho_{2}\left(x,y\right)=A_{3},\\ F_{i}\left(t,x,y,\omega_{1},\omega_{2}\right)={\left(x+e^{\lambda d}A_{3}y\right)}^{\tau }D_{i}\left(t\right)\left(x+e^{\lambda d}A_{3}y\right)+\omega_{1}^{\tau }R_{i1}\omega_{1}+\omega_{2}^{\tau }R_{i2}\omega_{2},i=1,2,\\ h_{i}\left(t,x,y,\omega_{1},\omega_{2}\right)={\left(x_{T}+e^{\lambda d}A_{3}y_{T}\right)}^{\tau }G_{i}\left(x_{T}+e^{\lambda d}A_{3}y_{T}\right),i=1,2,\\ \;\varsigma \left(t,x,y,\omega_{1},\omega_{2}\right)=A_{4}x+A_{5}y,\\ \;\nu \left(t,x,y,\omega_{1},\omega_{2}\right)=A_{6}x+A_{7}y. \end{array}\right. \end{array}$$

**Assumption 1.**  *Given the feasible strategy pairs $\omega_{1}\left(t\right)\in \Omega_{1}$ and $\omega_{2}\left(t\right)\in \Omega_{2}$, if $\left(X_{s},Y_{s}\right)$ follows $dX_{s}=\rho_{1}(s,X_{s},Y_{s},\omega_{1},$ $\omega_{2})ds+\rho_{2}\left(X_{s},Y_{s}\right)\varrho_{s}ds+\varsigma \left(s,X_{s},Y_{s},\omega_{1},\omega_{2}\right)dC_{s}+\nu \left(s,X_{s},Y_{s},\omega_{1},\omega_{2}\right)dV_{s}$, there exists an operator $Z\left(x,y\right):R^{n}\times R^{k}\to R^{n}$ such that*

(9) $$\begin{array}{c} e^{\lambda d}\Xi_{x}Z\left(x,y\right)\rho_{2}\left(x,y\right)-\Xi_{y}Z\left(x,y\right)=0,\;\forall \left(x,y\right)\in R^{n}\times R^{k}, \end{array}$$

*where $\Xi_{x}Z\left(x,y\right)$ and $\Xi_{y}Z\left(x,y\right)$ present the Jacobi matrices of Z(x,y) in x and in y, respectively.*

*Let $L=R\times y(C_{R}\left[-d,0\right]).$ For $\psi \in C_{R}\left[-d,0\right],$ we denote $x\left(\psi \right)=\psi \left(0\right),y\left(\psi \right)=\int_{-d}^{0}e^{\lambda s}F\left(\psi \left(s\right)\right)ds$, and ϱ(ψ)=F(ψ(−d)), where $F:R^{n}\to R^{k}$ is a differentiable function. Then, the new state process $Z_{t}=Z\left(X_{t},Y_{t}\right)\in Z\left(L\right).$*

*Define*

*$\tilde{\rho }:\left[0,+\infty \right)\times R^{n}\times R^{k}\times \Omega_{1}\times \Omega_{2}\to R^{n}$ by*

$$\tilde{\rho }\left(t,x,y,\omega_{1},\omega_{2}\right)=\Xi_{x}Z\left(x,y\right)\rho_{1}\left(t,x,y,\omega_{1},\omega_{2}\right)+\Xi_{y}Z\left(x,y\right)\left(F\left(x\right)-\lambda y\right),$$

$\tilde{\varsigma }:\left[0,+\infty \right)\times R^{n}\times R^{k}\times \Omega_{1}\times \Omega_{2}\to R^{n\times l}$ *by*

$$\tilde{\varsigma }\left(t,x,y,\omega_{1},\omega_{2}\right)=\Xi_{x}Z\left(x,y\right)\varsigma \left(t,x,y,\omega_{1},\omega_{2}\right),$$

*and $\tilde{\nu }:\left[0,+\infty \right)\times R^{n}\times R^{k}\times \Omega_{1}\times \Omega_{2}\to R^{n\times l}$ by*

$$\tilde{\nu }\left(t,x,y,\omega_{1},\omega_{2}\right)=\Xi_{x}Z\left(x,y\right)\nu \left(t,x,y,\omega_{1},\omega_{2}\right).$$

*If the functions $\tilde{\rho }$, $\tilde{\varsigma }$, $\tilde{\nu }$, $F_{i}$, and $h_{i}$ by Z(x,y) transform, then a finite-dimensional model can be obtained.*

**Assumption 2.**  *There are functions*

(10) $$\begin{array}{cc} & \bar{\rho }:\left[0,+\infty \right)\times R^{n}\times \Omega_{1}\times \Omega_{2}\to R^{n},\;\bar{\varsigma }:\left[0,+\infty \right)\times R^{n}\times \Omega_{1}\times \Omega_{2}\to R^{n\times l},\\ & \;\bar{\nu }:\left[0,+\infty \right)\times R^{n}\times \Omega_{1}\times \Omega_{2}\to R^{n\times l},\bar{F_{i}}:\left[0,+\infty \right)\times R^{n}\times \Omega_{1}\times \Omega_{2}\to R,\;\bar{h_{i}}:R^{n}\to R,i=1,2. \end{array}$$

*such that for all $t\in \left[0,T\right],\omega_{1}\in \Omega_{1},\omega_{2}\in \Omega_{2},\left(x,y\right)\in R^{n}\times R^{k},$ and it holds that*

(11) $$\begin{array}{cc} \bar{\rho }\left(t,Z\left(x,y\right),\omega_{1},\omega_{2}\right)= & \tilde{\rho }\left(t,x,y,\omega_{1},\omega_{2}\right),\;\bar{\varsigma }\left(t,Z\left(x,y\right),\omega_{1},\omega_{2}\right)=\tilde{\varsigma }\left(t,x,y,\omega_{1},\omega_{2}\right),\\ \;\bar{\nu }\left(t,Z\left(x,y\right),\omega_{1},\omega_{2}\right)= & \tilde{\nu }\left(t,x,y,\omega_{1},\omega_{2}\right),\\ \bar{F_{i}}\left(t,Z\left(x,y\right),\omega_{1},\omega_{2}\right)= & F_{i}\left(t,x,y,\omega_{1},\omega_{2}\right),\;\bar{h_{i}}\left(Z\left(x,y\right)\right)=h_{i}\left(x,y\right). \end{array}$$

*Now, we can transform the infinite-dimensional differential Nash equilibrium problems (6) and (7) into finite-dimensional differential Nash equilibrium problems by Z. For $\varphi_{t}\in C_{R}\left[-d,0\right],$ define $z=Z\left(x\left(\varphi_{t}\right),y\left(\varphi_{t}\right)\right)\in Z\left(L\right).$ Then, for t∈[0,T], the Nash equilibrium problem can be transformed into the following (12)–(14).*

(12) $$\begin{array}{c} \left\{\begin{array}{c} {\hat{J}}_{1}\left(\omega_{1}\left(t\right),\omega_{2}\left(t\right)\right)={\bar{J}}_{1}\left(t,z\right)=\underset{\omega_{1}\left(t\right)\in \Omega_{1}}{\sup}{\left\{\int_{t}^{T}{\bar{F}}_{1}\left(s,z_{s},\omega_{1}\left(s\right),\omega_{2}\left(s\right)\right)ds+{\bar{h}}_{1}\left(Z_{T}\right)\right\}}_{\sup}\left(\beta \right),\\ {\hat{J}}_{2}\left(\omega_{1}\left(t\right),\omega_{2}\left(t\right)\right)={\bar{J}}_{2}\left(t,z\right)=\underset{\omega_{2}\left(t\right)\in \Omega_{2}}{\sup}{\left\{\int_{t}^{T}{\bar{F}}_{2}\left(s,z_{s},\omega_{1}\left(s\right),\omega_{2}\left(s\right)\right)ds+{\bar{h}}_{2}\left(Z_{T}\right)\right\}}_{\sup}\left(\beta \right), \end{array}\right. \end{array}$$

(13) $$\begin{array}{c} \left\{\begin{array}{c} {\hat{J}}_{1}\left(\omega_{1}^{*}\left(t\right),\omega_{2}^{*}\left(t\right)\right)\geq {\hat{J}}_{1}\left(\omega_{1}\left(t\right),\omega_{2}^{*}\left(t\right)\right),\forall \omega_{1}\left(t\right)\in \Omega_{1},\\ {\hat{J}}_{2}\left(\omega_{1}^{*}\left(t\right),\omega_{2}^{*}\left(t\right)\right)\geq {\hat{J}}_{2}\left(\omega_{1}^{*}\left(t\right),\omega_{2}\left(t\right)\right),\forall \omega_{2}\left(t\right)\in \Omega_{2}, \end{array}\right. \end{array}$$

(14) $$\begin{array}{c} \left\{\begin{array}{c} dZ_{s}=\bar{\rho }\left(s,Z_{s},\omega_{1}\left(s\right),\omega_{2}\left(s\right)\right)ds+\bar{\varsigma }\left(s,Z_{s},\omega_{1}\left(s\right),\omega_{2}\left(s\right)\right)dC_{s}+\bar{\nu }\left(s,Z_{s},\omega_{1}\left(s\right),\omega_{2}\left(s\right)\right)dV_{s},\\ Z_{t}=z,\\ \omega_{1}\left(s\right)\in \Omega_{1},\omega_{2}\left(s\right)\in \Omega_{2},\;s\in \left[t,T\right]. \end{array}\right. \end{array}$$

**Theorem 1.**  *If $A_{2}\left(t\right)=e^{\lambda d}\left(\lambda I+A_{1}\left(t\right)+e^{\lambda d}A_{3}\left(t\right)\right)A_{3}\left(t\right)$, $A_{5}\left(t\right)=e^{\lambda d}A_{4}\left(t\right)A_{3}\left(t\right)$, $A_{7}\left(t\right)=e^{\lambda d}A_{6}\left(t\right)A_{3}\left(t\right)$ hold in an uncertain delay linear quadratic differential game model with jumps (3), then the Nash equilibrium strategies are*

(15) $$\begin{array}{c} \left\{\begin{array}{c} \omega_{1}^{*}\left(t\right)=-R_{11}^{-1}B_{1}^{\tau }Q_{1}\left(t\right)z\\ \omega_{2}^{*}\left(t\right)=-R_{22}^{-1}B_{2}^{\tau }Q_{2}\left(t\right)z \end{array}\right. \end{array}$$

*and the optimal value of (3) with initial value $Z_{0}=z_{0}$ is*

$$J_{1}\left(0,\varphi_{0}\right)={\left(z_{0}^{*}\right)}^{\tau }Q_{1}\left(0\right)z_{0}^{*},J_{2}\left(0,\varphi_{0}\right)={\left(z_{0}^{*}\right)}^{\tau }Q_{2}\left(0\right)z_{0}^{*}$$

*where $z=x+e^{\lambda d}A_{3}y$, and $Q_{1}$ and $Q_{2}$ satisfy the following Riccati equation.*

(16) $$\begin{array}{c} \left\{\begin{array}{c} {\dot{Q}}_{1}\left(t\right)=-\{D_{1}-Q_{1}^{\tau }B_{1}R_{11}^{-1}B_{1}^{\tau }Q_{1}+Q_{2}^{\tau }B_{2}R_{22}^{-1}R_{12}R_{22}^{-1}B_{2}^{\tau }Q_{2}+Q_{1}^{\tau }(A_{1}+e^{\lambda d}A_{3}+\frac{\sqrt{3}}{\pi }\ln\frac{1-\beta }{\beta }A_{4}\\ \;\;\;\;+kA_{6})+{\left(A_{1}+e^{\lambda d}A_{3}+\frac{\sqrt{3}}{\pi }\ln\frac{1-\beta }{\beta }A_{4}+kA_{6}\right)}^{\tau }Q_{1}-2Q_{1}^{\tau }B_{2}R_{22}^{-1}B_{2}^{\tau }Q_{2}\},\;\mathrm{if}\;\left(t,z\right)\in \Delta_{1}\\ {\dot{Q}}_{1}\left(t\right)=-\{D_{1}-Q_{1}^{\tau }B_{1}R_{11}^{-1}B_{1}^{\tau }Q_{1}+Q_{2}^{\tau }B_{2}R_{22}^{-1}R_{12}R_{22}^{-1}B_{2}^{\tau }Q_{2}+Q_{1}^{\tau }(A_{1}+e^{\lambda d}A_{3}-\frac{\sqrt{3}}{\pi }\ln\frac{1-\beta }{\beta }A_{4}\\ \;\;\;\;+kA_{6})+{\left(A_{1}+e^{\lambda d}A_{3}-\frac{\sqrt{3}}{\pi }\ln\frac{1-\beta }{\beta }A_{4}+kA_{6}\right)}^{\tau }Q_{1}-2Q_{1}^{\tau }B_{2}R_{22}^{-1}B_{2}^{\tau }Q_{2}\},\;\mathrm{if}\;\left(t,z\right)\in \Delta_{2}\\ Q_{1}\left(T\right)=G_{1}\\ {\dot{Q}}_{2}\left(t\right)=-\{D_{2}-Q_{2}^{\tau }B_{2}R_{22}^{-1}B_{2}^{\tau }Q_{2}+Q_{1}^{\tau }B_{1}R_{11}^{-1}R_{21}R_{11}^{-1}B_{1}^{\tau }Q_{1}+Q_{2}^{\tau }(A_{1}+e^{\lambda d}A_{3}+\frac{\sqrt{3}}{\pi }\ln\frac{1-\beta }{\beta }A_{4}\\ \;\;\;\;+kA_{6})+{\left(A_{1}+e^{\lambda d}A_{3}+\frac{\sqrt{3}}{\pi }\ln\frac{1-\beta }{\beta }A_{4}+kA_{6}\right)}^{\tau }Q_{2}-2Q_{2}^{\tau }B_{1}R_{11}^{-1}B_{1}^{\tau }Q_{1}\},\;\mathrm{if}\;\left(t,z\right)\in \Delta_{1}\\ {\dot{Q}}_{2}\left(t\right)=-\{D_{2}-Q_{2}^{\tau }B_{2}R_{22}^{-1}B_{2}^{\tau }Q_{2}+Q_{1}^{\tau }B_{1}R_{11}^{-1}R_{21}R_{11}^{-1}B_{1}^{\tau }Q_{1}+Q_{2}^{\tau }(A_{1}+e^{\lambda d}A_{3}-\frac{\sqrt{3}}{\pi }\ln\frac{1-\beta }{\beta }A_{4}\\ \;\;\;\;+kA_{6})+{\left(A_{1}+e^{\lambda d}A_{3}-\frac{\sqrt{3}}{\pi }\ln\frac{1-\beta }{\beta }A_{4}+kA_{6}\right)}^{\tau }Q_{2}-2Q_{2}^{\tau }B_{1}R_{11}^{-1}B_{1}^{\tau }Q_{1}\},\;\mathrm{if}\;\left(t,z\right)\in \Delta_{2}\\ Q_{2}\left(T\right)=G_{2} \end{array}\right. \end{array}$$

*where*

*(1)*  *if $A_{6}\left(t\right)\geq 0$, then $k=1-\frac{\beta }{2(1-\vartheta_{2})}\left(0<\beta <1-\vartheta_{2}\right)$ or $k=\frac{1}{2}\left(1-\vartheta_{2}\leq \beta <1-\vartheta_{1}\right)$ or $k=\frac{1-\beta }{2\vartheta_{1}}\left(1-\vartheta_{1}\leq \beta <1\right)$;*
*(2)*  *if $A_{6}\left(t\right)<0$, then $k=\frac{\beta }{2\vartheta_{1}}\left(0<\beta \leq \vartheta_{1}\right)$ or $k=\frac{1}{2}\left(\vartheta_{1}<\beta \leq \vartheta_{2}\right)$ or $k=1-\frac{1-\beta }{2(1-\vartheta_{2})}\left(\vartheta_{2}<\beta <1\right)$.*
$$\begin{array}{cc} \Delta_{1}= & \left\{\left(t,z\right)|z^{\tau }Q_{i}\left(t\right)A_{4}\left(t\right)z\geq 0,\left(t,z\right)\in \left[0,T\right]\times {\left[a,b\right]}^{n}\right\},\\ \Delta_{2}= & \left\{\left(t,z\right)|z^{\tau }Q_{i}\left(t\right)A_{4}\left(t\right)z<0,\left(t,z\right)\in \left[0,T\right]\times {\left[a,b\right]}^{n}\right\}. \end{array}$$

See Appendix A for the proof process.

## 4. A Linear Quadratic Game Model Under Uncertain Jump and Delay Environment

In two-person uncertain Nash differential games, when the sum of the two players’ cost function is zero, i.e., $J_{1}=-J_{2}$, the game degenerates to a two-person zero-sum uncertain differential game problem. In this section, we consider the saddle point problem of a linear quadratic two-person zero-sum game model under an uncertain jump and delay environment. The uncertain jump and delay dynamic system is as follows:

(17) $$\begin{array}{c} \left\{\begin{array}{c} \;dX_{s}=\left\{A_{1}\left(s\right)X_{s}+A_{2}\left(s\right)Y_{s}+A_{3}\varrho_{s}+B_{1}\left(s\right)\omega_{1}\left(s\right)+B_{2}\left(s\right)\omega_{2}\left(s\right)\right\}ds+\{A_{4}\left(s\right)X_{s}\\ \;\;\;\;+A_{5}\left(s\right)Y_{s}\}dC_{s}+\left\{A_{6}\left(s\right)X_{s}+A_{7}\left(s\right)Y_{s}\right\}dV_{s},\;s\in \left[t,T\right]\\ \;Y_{s}=\int_{-d}^{0}e^{\lambda u}X_{s+u}du,\;\varrho_{s}=X_{s-d},\;s\in \left[t,T\right]\\ \;X_{s}=\varphi_{t}\left(s-t\right),\;t-d\leq s\leq t. \end{array}\right. \end{array}$$

In the above equation, $\omega_{1}\in \Omega_{1}\left[t,T\right]$, $\omega_{2}\in \Omega_{2}\left[t,T\right]$ are the control functions or decision functions. The admissible control sets are defined by $\Omega_{i}\left[t,T\right]=\left\{\omega_{i}:\left[t,T\right]\to \Omega_{i}\right|\omega_{i}$, which is a measurable function, where $\omega_{1}\subset R^{p}$ and $\omega_{2}\subset R^{q}$ are nonempty closed sets. $X_{s},A_{1}\left(s\right),A_{2}\left(s\right),A_{3},B_{1}\left(s\right),B_{1}\left(s\right),A_{4}\left(s\right),A_{5}\left(s\right),A_{6}\left(s\right),A_{7}\left(s\right),d,\lambda ,\varphi_{t}$, $C_{t}={\left(C_{t1},C_{t2},\cdots ,C_{tl}\right)}^{\tau }$, and $V_{t}={\left(V_{t1},V_{t2},\cdots ,V_{tl}\right)}^{\tau }$ are defined the same as in Model (3). For any 0<t<T and some given confidence level β∈(0,1), we choose the objective function as follows:

(18) $$\begin{array}{c} J\left(t,\varphi_{t}\right)=J\left(\omega_{1},\omega_{2}\right)={\left\{\int_{t}^{T}F\left(s,X_{s},Y_{s},\omega_{1},\omega_{2}\right)ds+h\left(X_{T},T\right)\right\}}_{\sup}\left(\beta \right), \end{array}$$

where $F:\left[0,T\right]\times R^{n}\times R^{k}\times R^{p}\times R^{q}\to R$ is the continuous function of state and control, and $h:\left[0,T\right]\times R^{n}\to R$ is the continuous function of terminal reward. If there exists a pair $\left({\omega_{1}}^{*},{\omega_{2}}^{*}\right)$ satisfying

$$J({\omega_{1}}^{*},\omega_{2})\leq J({\omega_{1}}^{*},{\omega_{2}}^{*})\leq J(\omega_{1},{\omega_{2}}^{*}),$$

then the pair $\left({\omega_{1}}^{*},{\omega_{2}}^{*}\right)$ are called the saddle point of the zero-sum uncertain differential game. 

We take $J(\omega_{1},\omega_{2})$ as follows.

(19) $$\begin{array}{c} J\left(\omega_{1},\omega_{2}\right)={\left\{\frac{1}{2}\int_{0}^{T}\left({\left(X_{s}+e^{\lambda d}A_{3}Y_{s}\right)}^{\tau }P\left(s\right)\left(X_{s}+e^{\lambda d}A_{3}Y_{s}\right)+\omega_{2}^{\tau }G\left(s\right)\omega_{2}-\omega_{1}^{\tau }H\left(s\right)\omega_{1}\right)ds+X_{T}^{\tau }\Psi X_{T}\right\}}_{\sup}\left(\beta \right), \end{array}$$

where P(s),G(s),H(s),Ψ are appropriate size matrix functions, and P(s) is the symmetric nonnegative definite matrix, and G(s),H(s) are the symmetric positive definite matrix.

**Theorem 2.**  For the saddle equilibrium problem (17), if $A_{2}\left(t\right)=e^{\lambda d}\left(\lambda I+A_{1}\left(t\right)+e^{\lambda d}A_{3}\left(t\right)\right)A_{3}\left(t\right)$, $A_{5}\left(t\right)=e^{\lambda d}A_{4}\left(t\right)A_{3}\left(t\right)$, $A_{7}\left(t\right)=e^{\lambda d}A_{6}\left(t\right)A_{3}\left(t\right)$ hold and Q(t) is the solution of the following Riccati differential Equation (20), then the control strategy pair $\left(\omega_{1}^{*},\omega_{2}^{*}\right)$ of (21) are the equilibrium solutions of problem (17), and the equilibrium value is

$$\begin{array}{c} J\left(0,x_{0}\right)=\frac{1}{2}z_{0}^{\tau }Q\left(0\right)z_{0}. \end{array}$$

(20) $$\begin{array}{c} \left\{\begin{array}{c} \dot{Q}\left(t\right)+{\left(A_{1}\left(t\right)+e^{\lambda d}A_{3}\right)}^{\tau }Q\left(t\right)+Q\left(t\right)\left(A_{1}\left(t\right)+e^{\lambda d}A_{3}\right)+P\left(t\right)+\frac{\sqrt{3}}{\pi }\ln\frac{1-\beta }{\beta }Q\left(t\right)A_{4}\left(t\right)\\ +\frac{\sqrt{3}}{\pi }\ln\frac{1-\beta }{\beta }A_{4}^{\tau }\left(t\right)Q\left(t\right)+kQ\left(t\right)A_{6}\left(t\right)+kA_{6}^{\tau }\left(t\right)Q\left(t\right)\\ +Q\left(t\right)B_{1}\left(t\right)H^{-1}\left(t\right)B_{1}^{\tau }\left(t\right)Q\left(t\right)-Q\left(t\right)B_{2}\left(t\right)G^{-1}\left(t\right)B_{2}^{\tau }\left(t\right)Q\left(t\right)=0,\;\;\mathrm{if}\;\left(t,z\right)\in \Delta_{1}\\ \dot{Q}\left(t\right)+{\left(A_{1}\left(t\right)+e^{\lambda d}A_{3}\right)}^{\tau }Q\left(t\right)+Q\left(t\right)\left(A_{1}\left(t\right)+e^{\lambda d}A_{3}\right)+P\left(t\right)-\frac{\sqrt{3}}{\pi }\ln\frac{1-\beta }{\beta }Q\left(t\right)A_{4}\left(t\right)\\ -\frac{\sqrt{3}}{\pi }\ln\frac{1-\beta }{\beta }A_{4}^{\tau }\left(t\right)Q\left(t\right)+kQ\left(t\right)A_{6}\left(t\right)+kA_{6}^{\tau }\left(t\right)Q\left(t\right)\\ +Q\left(t\right)B_{1}\left(t\right)H^{-1}\left(t\right)B_{1}^{\tau }\left(t\right)Q\left(t\right)-Q\left(t\right)B_{2}\left(t\right)G^{-1}\left(t\right)B_{2}^{\tau }\left(t\right)Q\left(t\right)=0,\;\;\mathrm{if}\;\left(t,z\right)\in \Delta_{2}\\ Q(T)=2\Psi , \end{array}\right. \end{array}$$

*and*

(21) $$\begin{array}{c} \omega_{1}^{*}=H^{-1}\left(t\right)B_{1}^{\tau }\left(t\right)Q\left(t\right)z,\omega_{2}^{*}=-G^{-1}\left(t\right)B_{2}^{\tau }\left(t\right)Q\left(t\right)z, \end{array}$$

*where $z=x+e^{\lambda d}A_{3}y$,*

*(1)*  *if $A_{6}\left(t\right)\geq 0$, then $k=1-\frac{\beta }{2(1-\vartheta_{2})}\left(0<\beta <1-\vartheta_{2}\right)$ or $k=\frac{1}{2}\left(1-\vartheta_{2}\leq \beta <1-\vartheta_{1}\right)$ or $k=\frac{1-\beta }{2\vartheta_{1}}\left(1-\vartheta_{1}\leq \beta <1\right)$;*
*(2)*  *if $A_{6}\left(t\right)<0$, then $k=\frac{\beta }{2\vartheta_{1}}\left(0<\beta \leq \vartheta_{1}\right)$ or $k=\frac{1}{2}\left(\vartheta_{1}<\beta \leq \vartheta_{2}\right)$ or $k=1-\frac{1-\beta }{2(1-\vartheta_{2})}\left(\vartheta_{2}<\beta <1\right)$.*
$$\begin{array}{cc} \Delta_{1}= & \left\{\left(t,z\right)|z^{\tau }Q\left(t\right)A_{4}\left(t\right)z\geq 0,\left(t,z\right)\in \left[0,T\right]\times {\left[a,b\right]}^{n}\right\},\\ \Delta_{2}= & \left\{\left(t,z\right)|z^{\tau }Q\left(t\right)A_{4}\left(t\right)z<0,\left(t,z\right)\in \left[0,T\right]\times {\left[a,b\right]}^{n}\right\}. \end{array}$$
  .

**Proof.**  Since the saddle point equilibrium of the problem (17) is a special case of Nash equilibrium of the problem (3), similar to the proof of Theorem 1, we end here. □

**Remark 2.**  *Comparing with the model in Ref. [20], we introduce jump factors and delays, and extend the model to the multidimensional case. In order to obtain Q(t), we need to judge the sign of $z^{\tau }Q\left(t\right)A_{4}\left(t\right)z$, which undoubtedly increases the difficulty of solving the problem. Further, when we know the positive or negative of $Q\left(t\right)A_{4}\left(t\right)$, then this problem is easier to solve. Therefore, we consider a necessary and sufficient condition for the existence of the saddle point of a special one-dimensional model that can be used in later application.*

(22) $$\begin{array}{c} \left\{\begin{array}{c} \;\mathrm{Find}\;(\omega_{1}^{*},\omega_{2}^{*})\;\mathrm{such}\;\;\mathrm{that}\;\\ J\left(\omega_{1},\omega_{2}^{*}\right)\leq J\left(\omega_{1}^{*},\omega_{2}^{*}\right)\leq J\left(\omega_{1}^{*},\omega_{2}\right)\\ \;\mathrm{subject}\;\;\mathrm{to}:\;\\ \;\;dX_{s}=\left\{A_{1}\left(s\right)X_{s}+A_{2}\left(s\right)Y_{s}+A_{3}\varrho_{s}+B_{1}\left(s\right)\omega_{1}\left(s\right)+B_{2}\left(s\right)\omega_{2}\left(s\right)\right\}ds+\{A_{4}\left(s\right)X_{s}\\ \;\;\;\;+A_{5}\left(s\right)Y_{s}\}dC_{s}+\left\{A_{6}\left(s\right)X_{s}+A_{7}\left(s\right)Y_{s}\right\}dV_{s},\;s\in \left[0,T\right]\\ \;Y_{s}=\int_{-d}^{0}e^{\lambda u}X_{s+u}du,\;\varrho_{s}=X_{s-d},\;s\in \left[0,T\right]\\ \;X_{s}=\varphi_{0}\left(s\right),\;-d\leq s\leq 0, \end{array}\right. \end{array}$$

where

(23) $$\begin{array}{cc} & J\left(0,\varphi_{0}\right)=J\left(\omega_{1},\omega_{2}\right)=\\ & {\left\{\frac{1}{2}\int_{0}^{T}\left(P\left(s\right){\left(X_{s}+e^{\lambda d}A_{3}Y_{s}\right)}^{2}+G\left(s\right)\omega_{2}^{2}-H\left(s\right)\omega_{1}^{2}\right)ds+\frac{1}{2}\Psi_{f}{\left(X_{T}+e^{\lambda d}A_{3}Y_{T}\right)}^{2}\right\}}_{\sup}\left(\beta \right). \end{array}$$

In the above model, P(s)≥0, G(s)>0, H(s)>0, $A_{1}\left(s\right),$ $A_{2}\left(s\right),$ $B_{1}\left(s\right),$ $B_{2}\left(s\right),$ $A_{4}\left(s\right)>0$, $A_{5}\left(s\right)$, $A_{6}\left(s\right)\geq 0$, $A_{7}\left(s\right)$ are continuous bounded functions, and $A_{3}$ and $\Psi_{f}$ are the given constants. $J(0,\varphi_{0})$ represents the equilibrium reward obtainable in [0,T]. 

We note that the value functions $J(0,\varphi_{0})$ in (23) were defined in an infinite-dimensional space, so we will use the idea of Section 3 to transform the problem into a finite-dimensional space by constructing a new state Z. For this purpose, we adopt the above Assumption 1 again. For Questions (22) and (23), we denote that

(24) $$\begin{array}{c} \left\{\begin{array}{c} \;\rho_{1}\left(t,x,y,\omega_{1},\omega_{2}\right)=A_{1}x+A_{2}y+B_{1}\omega_{1}+B_{2}\omega_{2},\\ \;\rho_{2}\left(x,y\right)=A_{3},\\ \;F\left(t,x,y,\omega_{1},\omega_{2}\right)=P{\left(x+e^{\lambda d}A_{3}y\right)}^{2}+G\omega_{2}^{2}-H\omega_{1}^{2},\\ \;h\left(t,x,y,\omega_{1},\omega_{2}\right)=\frac{1}{2}\Psi_{f}{\left(x+e^{\lambda d}A_{3}y\right)}^{2},\\ \;\varsigma \left(t,x,y,\omega_{1},\omega_{2}\right)=A_{4}x+A_{5}y,\\ \;\nu \left(t,x,y,\omega_{1},\omega_{2}\right)=A_{6}x+A_{7}y. \end{array}\right. \end{array}$$

Define $\tilde{\rho }:\left[0,+\infty \right)\times R\times R\times \Omega_{1}\times \Omega_{2}\to R$ by

$$\tilde{\rho }\left(t,x,y,\omega_{1},\omega_{2}\right)=Z_{x}\left(x,y\right)\rho_{1}\left(t,x,y,\omega_{1},\omega_{2}\right)+Z_{y}\left(x,y\right)\left(F\left(x\right)-\lambda y\right),$$

$\tilde{\varsigma }:\left[0,+\infty \right)\times R\times R\times \Omega_{1}\times \Omega_{2}\to R$ by

$$\tilde{\varsigma }\left(t,x,y,\omega_{1},\omega_{2}\right)=Z_{x}\left(x,y\right)\varsigma \left(t,x,y,\omega_{1},\omega_{2}\right),$$

and $\tilde{\nu }:\left[0,+\infty \right)\times R\times R\times \Omega_{1}\times \Omega_{2}\to R$ by

$$\tilde{\nu }\left(t,x,y,\omega_{1},\omega_{2}\right)=Z_{x}\left(x,y\right)\nu \left(t,x,y,\omega_{1},\omega_{2}\right).$$

If the functions $\tilde{\rho }$, $\tilde{\varsigma }$, and $\tilde{\nu }$, *F* as well as *h* depend on (x,y) through Z(x,y) only, then the problem could be reduced to a finite-dimensional problem.

**Assumption 3.**  *There are functions*

(25) $$\begin{array}{cc} & \;\;\;\bar{\rho }:\left[0,+\infty \right)\times R\times \Omega_{1}\times \Omega_{2}\to R,\;\bar{\varsigma }:\left[0,+\infty \right)\times R\times \Omega_{1}\times \Omega_{2}\to R,\\ & \;\bar{\nu }:\left[0,+\infty \right)\times R\times \Omega_{1}\times \Omega_{2}\to R,\bar{F}:\left[0,+\infty \right)\times R\times \Omega_{1}\times \Omega_{2}\to R,\;\bar{h}:R\to R. \end{array}$$

*such that for all $t\in \left[0,T\right],\omega_{1}\in \Omega_{1},\omega_{2}\in \Omega_{2},\left(x,y\right)\in R\times R,$ it holds that*

(26) $$\begin{array}{cc} \bar{\rho }\left(t,Z\left(x,y\right),\omega_{1},\omega_{2}\right)= & \tilde{\rho }\left(t,x,y,\omega_{1},\omega_{2}\right),\;\bar{\varsigma }\left(t,Z\left(x,y\right),\omega_{1},\omega_{2}\right)=\tilde{\varsigma }\left(t,x,y,\omega_{1},\omega_{2}\right),\\ \;\bar{\nu }\left(t,Z\left(x,y\right),\omega_{1},\omega_{2}\right)= & \tilde{\nu }\left(t,x,y,\omega_{1},\omega_{2}\right),\\ \bar{F}\left(t,Z\left(x,y\right),\omega_{1},\omega_{2}\right)= & F\left(t,x,y,\omega_{1},\omega_{2}\right),\;\bar{h}\left(Z\left(x,y\right)\right)=h\left(x,y\right). \end{array}$$

*Now, we can transform the infinite-dimensional saddle point equilibrium problems into finite-dimensional saddle point equilibrium problems by Z. For $\varphi_{t}\in C_{R}\left[-d,0\right],$ define $z=Z\left(x\left(\varphi_{t}\right),y\left(\varphi_{t}\right)\right)\in Z\left(L\right).$ Then, for t∈[0,T], the saddle equilibrium problem of the one-dimensional case of the objective function (18) and model (22) is equivalent to solving (27)–(29).*

(27) $$\begin{array}{c} \hat{J}\left(\omega_{1}\left(t\right),\omega_{2}\left(t\right)\right)=\bar{J}\left(t,z\right)={\left\{\int_{t}^{T}\bar{F}\left(s,z_{s},\omega_{1}\left(s\right),\omega_{2}\left(s\right)\right)ds+\bar{h}\left(Z_{T}\right)\right\}}_{\sup}\left(\beta \right) \end{array}$$

(28) $$\begin{array}{c} \hat{J}({\omega_{1}}^{*},\omega_{2})\leq \hat{J}({\omega_{1}}^{*},{\omega_{2}}^{*})\leq \hat{J}(\omega_{1},{\omega_{2}}^{*}), \end{array}$$

(29) $$\begin{array}{c} \left\{\begin{array}{c} dZ_{s}=\bar{\rho }\left(s,Z_{s},\omega_{1}\left(s\right),\omega_{2}\left(s\right)\right)ds+\bar{\varsigma }\left(s,Z_{s},\omega_{1}\left(s\right),\omega_{2}\left(s\right)\right)dC_{s}+\bar{\nu }\left(s,Z_{s},\omega_{1}\left(s\right),\omega_{2}\left(s\right)\right)dV_{s}\\ Z_{t}=z,\\ \omega_{1}\left(s\right)\in \Omega_{1},\omega_{2}\left(s\right)\in \Omega_{2},\;s\in \left[t,T\right] \end{array}\right. \end{array}$$

**Theorem 3.**  *If $A_{2}\left(t\right)=e^{\lambda d}\left(\lambda +A_{1}\left(t\right)+e^{\lambda d}A_{3}\right)A_{3}$, $A_{5}\left(t\right)=e^{\lambda d}A_{4}\left(t\right)A_{3}$, and $A_{7}\left(t\right)=e^{\lambda d}A_{6}\left(t\right)A_{3}$ hold in Model (22), then a necessary and sufficient condition for the equilibrium solutions to Problem (22) is*

$$\begin{array}{c} \omega_{1}^{*}=\frac{Q\left(t\right)B_{1}\left(t\right)}{H(t)}z,\omega_{2}^{*}=-\frac{Q\left(t\right)B_{2}\left(t\right)}{G(t)}z \end{array}$$

*where $z=x+e^{\lambda d}A_{3}y$, and Q(t) satisfies:*

$$\begin{array}{c} \left\{\begin{array}{c} \frac{dQ(t)}{dt}+2Q\left(t\right)\left(A_{1}\left(t\right)+e^{\lambda d}A_{3}\right)+P+\frac{Q^{2}\left(t\right)B_{1}^{2}\left(t\right)}{H(t)}-\frac{Q^{2}\left(t\right)B_{2}^{2}\left(t\right)}{G(t)}\\ \;\;\;\;\;\;+\frac{2\sqrt{3}}{\pi }\ln\frac{1-\beta }{\beta }Q\left(t\right)A_{4}\left(t\right)+2kA_{6}\left(t\right)Q\left(t\right)=0,\\ Q\left(T\right)=\Psi_{f}, \end{array}\right. \end{array}$$

*where $k=1-\frac{\beta }{2(1-\vartheta_{2})}\left(0<\beta <1-\vartheta_{2}\right)$ or $k=\frac{1}{2}\left(1-\vartheta_{2}\leq \beta <1-\vartheta_{1}\right)$ or $k=\frac{1-\beta }{2\vartheta_{1}}\left(1-\vartheta_{1}\leq \beta <1\right)$;*

*The equilibrium value is*

$$\begin{array}{c} J\left(0,x_{0}\right)=\frac{1}{2}Q\left(0\right){\left(x_{0}+e^{\lambda d}A_{3}\int_{-d}^{0}e^{\lambda u}X_{u}du\right)}^{2}. \end{array}$$

See Appendix A for the proof process.

## 5. Carbon Emission Reduction Problem

Delays and jumps are ubiquitous in economic and management issues, such as in consumption and carbon emission reduction. We will analyze a carbon emission reduction economic game model under an uncertain jump and delay environment. The problem is crucial for addressing global climate change and is widely studied in environmental economics, policy design, and sustainability sciences. A carbon emission reduction game problem involves modeling the strategic interactions between different players, such as countries, companies, or industries, who aim to reduce carbon emissions while balancing their economic interests. The problem typically focuses on how these players can optimize their strategies to achieve emission reduction targets, considering costs and regulatory policies. In the following carbon emission reduction model, $X_{t}$ represents the carbon-emitting resources stock of carbon-emitting enterprises, which not only represents the assets owned by carbon-emitting enterprises, but also includes a comprehensive quantified value of electricity, coal, oil, and natural gas. It is a widely recognized fact that the provision of essential energy resources such as electricity, coal, oil, and natural gas can experience delays. These delays may stem from a variety of causes including logistical challenges, infrastructure limitations, regulatory hurdles, and unpredictable events such as natural disasters or geopolitical conflicts. Thus, the assets are not only related to the time t, but also affected by $Y_{t}=\int_{-d}^{0}e^{\lambda s}X_{t+s}ds,\;\varrho_{t}=X_{t-d},\;t\geq 0$ at time t−d, and $\varphi_{t}\left(s\right)=X_{t+s},\;s\in \left[-d,0\right]$ indicates the assets of the time period [−d,0], λ are a constant. The growth rate of $X_{t}$ is a linear function of $X_{t}$, $Y_{t}$, carbon emissions, and government input measures. Moreover, despite the fact that governments and enterprises from various countries are actively taking measures to reduce carbon emissions, address global climate change, and promote sustainable development, the reality is often cruel. Certain groups or individuals, motivated by varied interests such as political gain, economic advantage, or other benefits, secretly endorse practices that increase carbon emissions. The influence of this part of “funding behind” cannot be ignored and therefore must be taken into account in analytical frameworks. However, their behavior is secretive and therefore not easily detected, and we cannot obtain sufficiently detailed data on their behavior. In this context, the expertise of criminal psychology professionals or specialists in carbon emission mitigation, particularly those focused on carbon policy, becomes invaluable. Their knowledge and experience are essential for evaluating the impact of this “funding behind” on the resources of carbon-emitting enterprises. In dealing with expert opinions, we adopt the uncertainty theory as a powerful tool, and consider that the carbon-emitting event itself is an emergency, so we describe the influence of “funding behind” as an uncertain jump process. In addition, considering the harmfulness and destructiveness of carbon-emitting enterprises, the average evaluation criterion of expected value is inappropriate for modeling the carbon emission reduction economic because it often ignores the interests of the majority of people. For the carbon emission reduction economic model, we believe that the optimistic value model is a relatively more appropriate choice because a certain confidence level (here, the confidence level is the success rate of the decision) can be selected, which also takes into account the interests of the majority. Thus, the model in a limited time horizon is as follows:

$$\begin{array}{c} \left\{\begin{array}{c} \;\mathrm{Find}\;(\omega_{1}^{*},\omega_{2}^{*})\;\mathrm{such}\;\;\mathrm{that}\;\\ J\left(\omega_{1},\omega_{2}^{*}\right)\leq J\left(\omega_{1}^{*},\omega_{2}^{*}\right)\leq J\left(\omega_{1}^{*},\omega_{2}\right)\\ \;\mathrm{subject}\;\;\mathrm{to}:\;\\ \;dX_{s}=\left(\epsilon X_{s}+e^{\lambda d}\left(e^{\lambda d}+\lambda +\epsilon \right)Y_{s}+\varrho_{s}+\omega_{1}-\omega_{2}\right)ds+\varsigma \left(X_{s}+e^{\lambda d}Y_{s}\right)dC_{s}\\ \;\;\;\;\;\;\;\;\;\;\;\;\;\;+\nu \left(X_{s}+e^{\lambda d}Y_{s}\right)dV_{s},\;s\in \left[0,T\right]\\ \;Y_{s}=\int_{-d}^{0}e^{\lambda u}X_{s+u}du,\;\varrho_{s}=X_{s-d},\;s\in \left[0,T\right]\\ \;X_{s}=\varphi_{0}\left(s\right),\;-d\leq s\leq 0, \end{array}\right. \end{array}$$

where

$$\begin{array}{c} J\left(\omega_{1},\omega_{2}\right)={\left\{\frac{1}{2}\lambda_{1}\int_{0}^{T}\left({\left(X_{s}+e^{\lambda d}Y_{s}\right)}^{2}+\omega_{2}^{2}-\omega_{1}^{2}\right)ds+\frac{1}{2}\lambda_{2}{\left(X_{T}+e^{\lambda d}Y_{T}\right)}^{2}\right\}}_{\sup}\left(\beta \right). \end{array}$$

where $\omega_{1}$ is the frequency of corporate carbon emissions and $\omega_{2}$ is government input measures. The performance index represents the economic losses caused by carbon emissions, $\lambda_{1},\lambda_{2}>0$ and $\omega_{1},\omega_{2}\geq 0,$ and ϵ, ς, and ν are the given positive constants. Let $Z\left(x,y\right)=x+e^{\lambda d}y;$ then, it is obvious that $e^{\lambda d}Z_{x}\left(x,y\right)-Z_{y}\left(x,y\right)=0$ holds, and that Assumption 1 holds. It is easy to see that Assumption 3 holds. Using Theorem 3, we can write the Riccati equation:

$$\begin{array}{c} \left\{\begin{array}{c} \dot{Q}+2\left(\epsilon +e^{\lambda d}\right)Q+\lambda_{1}+\frac{2\sqrt{3}}{\pi }\ln\frac{1-\beta }{\beta }\varsigma Q+2k\nu P=0,\\ Q\left(t\right)=\lambda_{2}, \end{array}\right. \end{array}$$

and we obtain

$$\begin{array}{cc} Q(t)= & -\frac{\lambda_{1}}{2\left(\epsilon +e^{\lambda d}\right)+\frac{2\sqrt{3}}{\pi }\ln\frac{1-\beta }{\beta }\varsigma +2k\nu }+\left(\lambda_{2}+\frac{\lambda_{1}}{2\left(\epsilon +e^{\lambda d}\right)+\frac{2\sqrt{3}}{\pi }\ln\frac{1-\beta }{\beta }\varsigma +2k\nu }\right)\\ & \exp\left(2\left(\epsilon +e^{\lambda d}\right)+\frac{2\sqrt{3}}{\pi }\ln\frac{1-\beta }{\beta }\varsigma +2k\nu \right)\left(T-t\right), \end{array}$$

where owing to ${\bar{J}}_{z}\left(\nu z\right)=\nu \geq 0$, then $k=1-\frac{\beta }{2(1-\vartheta_{2})}\left(0<\beta <1-\vartheta_{2}\right)$ or $k=\frac{1}{2}\left(1-\vartheta_{2}\leq \beta <1-\vartheta_{1}\right)$ or $k=\frac{1-\beta }{2\vartheta_{1}}\left(1-\vartheta_{1}\leq \beta <1\right)$.

The equilibrium strategy is obtained as follows:

$$\begin{array}{cc} \omega_{1}^{*}=\omega_{2}^{*}= & [-\frac{1}{2\left(\epsilon +e^{\lambda d}\right)+\frac{2\sqrt{3}}{\pi }\ln\frac{1-\beta }{\beta }\varsigma +2k\nu }+\left(\frac{\lambda_{2}}{\lambda_{1}}+\frac{\lambda_{1}}{2\left(\epsilon +e^{\lambda d}\right)+\frac{2\sqrt{3}}{\pi }\ln\frac{1-\beta }{\beta }\varsigma +2k\nu }\right)\\ & \exp(\left(2\left(\epsilon +e^{\lambda d}\right)+\frac{2\sqrt{3}}{\pi }\ln\frac{1-\beta }{\beta }\varsigma +2k\nu \right)\left(T-t\right))]z. \end{array}$$

The equilibrium values are given as follows:

$$\begin{array}{cc} J(t,x)= & \frac{1}{2}[-\frac{\lambda_{1}}{2\left(\epsilon +e^{\lambda d}\right)+\frac{2\sqrt{3}}{\pi }\ln\frac{1-\beta }{\beta }\varsigma +2k\nu }+\left(\lambda_{2}+\frac{\lambda_{1}}{2\left(\epsilon +e^{\lambda d}\right)+\frac{2\sqrt{3}}{\pi }\ln\frac{1-\beta }{\beta }\varsigma +2k\nu }\right)\cdot \\ & \exp\left(\left(2\left(\epsilon +e^{\lambda d}\right)+\frac{2\sqrt{3}}{\pi }\ln\frac{1-\beta }{\beta }\varsigma +2k\nu \right)\left(T-t\right)\right)]z^{2}, \end{array}$$

and

$$\begin{array}{cc} J(0,x_{0})= & \frac{1}{2}[-\frac{\lambda_{1}}{2\left(\epsilon +e^{\lambda d}\right)+\frac{2\sqrt{3}}{\pi }\ln\frac{1-\beta }{\beta }\varsigma +2k\nu }+\left(\lambda_{2}+\frac{\lambda_{1}}{2\left(\epsilon +e^{\lambda d}\right)+\frac{2\sqrt{3}}{\pi }\ln\frac{1-\beta }{\beta }\varsigma +2k\nu }\right)\cdot \\ & \exp\left(\left(2\left(\epsilon +e^{\lambda d}\right)+\frac{2\sqrt{3}}{\pi }\ln\frac{1-\beta }{\beta }\varsigma +2k\nu \right)T\right)]z_{0}^{2}, \end{array}$$

where $z=x_{t}+e^{\lambda d}y_{t}$, and $z_{0}=x_{0}+e^{\lambda d}y_{0}$. In order to explain the problem clearly, we take the following parameters:

$$x_{0}=10,T=3,\lambda =-20,d=0.2,\epsilon =0.05,\varsigma =1,\nu =0.3,\lambda_{1}=\lambda_{2}=1.$$

We let $\varphi_{0}\left(s\right)=\cos\pi s$, then $y_{0}=\int_{-0.2}^{0}e^{-20u}\cos\pi udu=\frac{\pi \sin\left(0.2\pi \right)+20\cos\left(0.2\pi \right)-20e^{-4}}{e^{-4}\left(\pi^{2}+400\right)}$ and $z_{0}=10+\frac{\pi \sin\left(0.2\pi \right)+20\cos\left(0.2\pi \right)-20e^{-4}}{\pi^{2}+400}$. We take $\vartheta_{1}=0.3,\;\vartheta_{2}=0.6$, and then we obtain the equilibrium value of different belief degree β. Meanwhile, we give the dynamic change curve of the equilibrium strategy $\left(\omega_{1}^{*},\omega_{2}^{*}\right)$ and equilibrium value J(t,x) of different belief degree β. According to Ref. [20], the equilibrium value $J(0,x_{0})$ without state delays and V-jumps process is

$$\begin{array}{cc} J(0,x_{0})= & \frac{1}{2}[-\frac{\lambda_{1}}{2\epsilon +\frac{2\sqrt{3}}{\pi }\ln\frac{1-\beta }{\beta }\varsigma }+\left(\lambda_{2}+\frac{\lambda_{1}}{2\epsilon +\frac{2\sqrt{3}}{\pi }\ln\frac{1-\beta }{\beta }\varsigma }\right)\cdot \\ & \exp\left(\left(2\epsilon +\frac{2\sqrt{3}}{\pi }\ln\frac{1-\beta }{\beta }\varsigma \right)T\right)]x_{0}^{2}, \end{array}$$

By taking the same parameters, we give the equilibrium value of various belief degrees β without state delays and the V-jumps process as the comparison.

To study the influence of parametric errors on the carbon emission reduction model, let us take the parameter β as an example. Based on the equilibrium value and error curves analysis, we firstly performed a sensitivity analysis; we perturbed the parameter β by 10% to examine the effect of this change on the equilibrium values both with and without delays. The results indicate that the equilibrium values are highly sensitive to changes in β, especially in the lower β range, where the variation is more pronounced.The analysis shows that in the “no delay” case, the equilibrium values exhibit more dramatic fluctuations in response to changes in β compared to the “with delays” case. The sensitivity curves demonstrate that the model reacts differently to parameter perturbations depending on whether state delays are included or not.

Then, we performed an error propagation analysis to assess the impact of parameter uncertainty (e.g., β ± 5%) on the equilibrium values. The results display the error bounds (upper and lower limits) for both the “no delay” and “with delays” cases.The “no delay” scenario shows larger error bounds compared to the “with delays” case, indicating greater sensitivity to parameter errors. In contrast, the “with delays” model exhibits more robustness to parameter errors, with smaller variations in equilibrium values.

**Figure 1:**
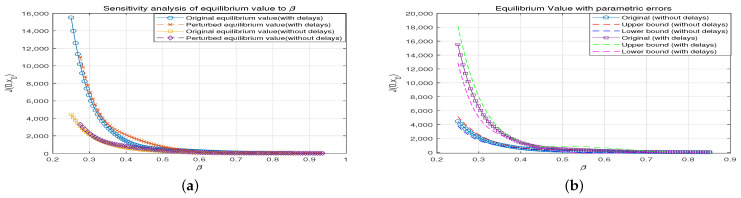
The equilibrium value. (**a**) Sensitivity analysis of equilibrium value. (**b**) Equilibrium value with parametric errors.

Figure 1a illustrates the smooth equilibrium values for both the “no delay” and “with delays” scenarios as functions of β. As β increases, the equilibrium values decrease in both cases, but the decline is sharper in the “no delay” case. The graph also presents the comparison between the original equilibrium values and those after a 10% perturbation in β. The perturbed curves deviate more noticeably from the original in the “no delay” case, indicating higher sensitivity to parameter changes. Figure 1b displays the equilibrium values with a ±5% parameter error. The error bounds are plotted as smooth curves above and below the original equilibrium values. In the “no delay” scenario, the error bounds are wider, reflecting a higher sensitivity to parameter uncertainty. In contrast, the “with delays” scenario has narrower error bounds, indicating more stability and resistance to parameter error.

Through sensitivity and error propagation analysis, it is evident that the “no delay” model is more sensitive to parameter changes and errors compared to the “with delays” model. This suggests that the “with delays” model demonstrates better robustness in the face of parameter uncertainties. The influence of β on the equilibrium values is particularly significant in the lower range. The error analysis reveals that parameter errors can have a substantial impact on model predictions, especially in the “no delay” case.

**Figure 2:**
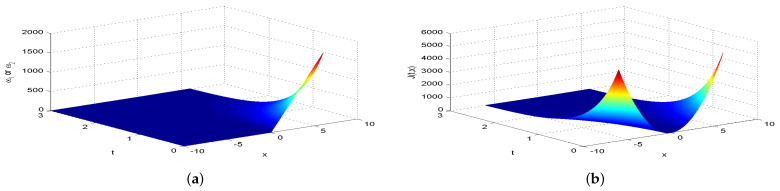
The equilibrium strategy value (**a**) and the equilibrium value (**b**) when β=0.25.

**Figure 3:**
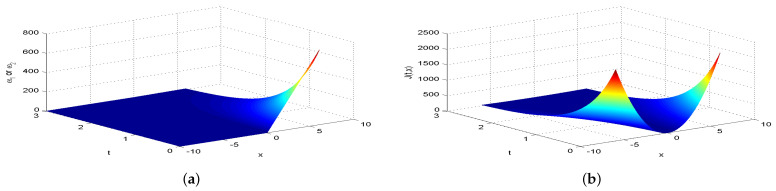
The equilibrium strategy value (**a**) and the equilibrium value (**b**) when β=0.30.

**Figure 4:**
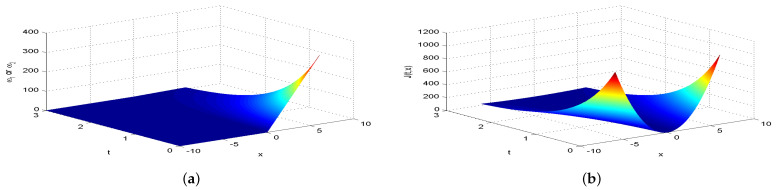
The equilibrium strategy value (**a**) and the equilibrium value (**b**) when β=0.35.

**Figure 5:**
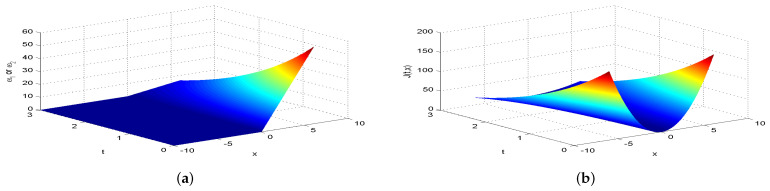
The equilibrium strategy value (**a**) and the equilibrium value (**b**) when β=0.50.

**Figure 6:**
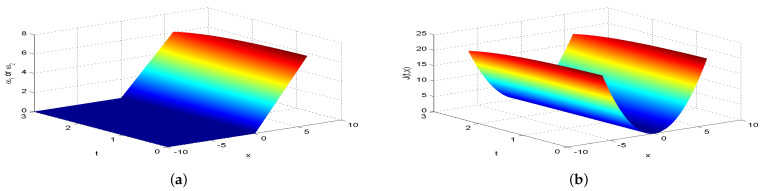
The equilibrium strategy value (**a**) and the equilibrium value (**b**) when β=0.75.

**Figure 7:**
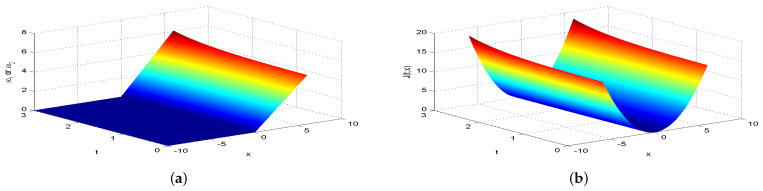
The equilibrium strategy value (**a**) and the equilibrium value (**b**) when β=0.80.

**Figure 8:**
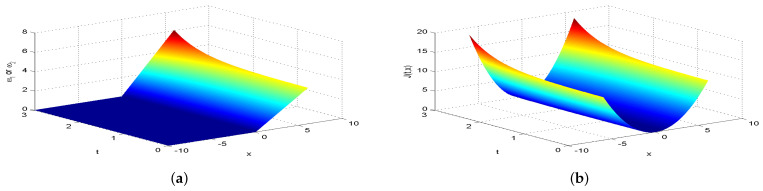
The equilibrium strategy value (**a**) and the equilibrium value (**b**) when β=0.85.

Next, we present Figure 2, Figure 3, Figure 4, Figure 5, Figure 6, Figure 7 and Figure 8, which intuitively illustrate the equilibrium strategy values and the equilibrium values.

Table 1 shows that the belief degree β represents the confidence level in reducing economic losses caused by carbon emissions. For instance, if carbon-emitting enterprises require an initial start-up fund of 10 million, they should prepare $10+\frac{\pi \sin\left(0.2\pi \right)+20\cos\left(0.2\pi \right)-20e^{-4}}{\pi^{2}+400}$ million dollars considering delays. From Table 1, we observe that the equilibrium value $J(0,x_{0})=15,523.0$ when β=0.25, resulting in government losses of 15,523.0 million. When β=0.30, the equilibrium value is 6493.70, and the government losses are 6493.7 million. At β=0.35, the equilibrium value is 2978.5, with corresponding government losses of 2978.5 million. When β=0.5, the equilibrium value drops to 499.4, and the losses are 499.4 million. At β=0.75, the equilibrium value further decreases to 60.2, with losses of 60.2 million. For β=0.80, the equilibrium value is 42.5, and the losses are 42.5 million. Finally, at β=0.85, the equilibrium value is 31.2, resulting in the smallest government losses of 31.2 million. This analysis reveals that both the government and carbon-emitting enterprises incur significant costs to achieve their objectives. It is also evident that due to the influence of jumps and delays, economic losses from terrorism are lower when β>0.5 and higher when β<0.5. Among the given belief degrees, β=0.85 results in the least economic loss, and generally, higher β values correlate with lower economic losses $J(0,x_{0})$. Additionally, the presence of jumps and delays increases the equilibrium value compared to scenarios without these factors when β∈(0,1). This means that economic losses rise due to jumps and delays, aligning with the real-world dynamics and the objective laws of counter-terrorism economic issues. From Figure 2, Figure 3, Figure 4, Figure 5, Figure 6, Figure 7 and Figure 8, we can intuitively observe the dynamic variation curves of the equilibrium strategy $\left(\omega_{1}^{*},\omega_{2}^{*}\right)$ and the equilibrium value J(t,x) at different confidence levels β. For governments, incorrect belief degrees may result in uneven resource allocation. For enterprises, mistakes in belief degrees can harm brand image and even lead to bankruptcy. Thus, improving the decision-making belief degree is crucial. Both governments and carbon-emitting enterprises must enhance the scientific and rational aspects of their decision-making processes, thoroughly considering various factors, conducting comprehensive research, and avoiding hasty decisions based on subjective assumptions. Establishing sound decision-making mechanisms and responsibility systems is also essential to ensure transparency, accountability, and support for sustainable economic and social development. In future development, governments and carbon-emitting enterprises should prioritize enhancing decision-making belief degrees by developing policies or strategies that both minimize emissions and maintain competitiveness and economic viability. Incorporating detailed economic analysis and being open to policy adjustments based on those insights will ensure that decisions are not only environmentally sound but also economically sustainable.

## 6. Conclusions

This study addressed the differential game optimistic value model for uncertain delay systems with jumps. By transforming the model into a finite-dimensional framework, the research provided valuable insights into the Nash equilibrium solution specifically for uncertain delay differential game optimistic value control models that incorporate jumps. Furthermore, the study identified the saddle equilibrium solution within a linear quadratic differential game optimistic value model under conditions characterized by uncertainty, delays and sudden jumps. To illustrate these concepts in a practical context, an example of a carbon emission reduction game was presented, highlighting the implications of the theoretical findings in real-world scenarios. This example not only demonstrated the applicability of the developed models but also underscored the critical role of strategic decision making in addressing pressing environmental challenges. Looking ahead, future research could delve deeper into both theoretical and practical dimensions. On the theoretical side, exploring more complex game structures involving singular systems, switched systems, or other non-linear systems could enhance our understanding of strategic interactions in uncertain environments. Investigating alternative criteria for decision making beyond the optimistic value might also yield new insights into equilibrium concepts and stability conditions. In terms of practical applications, expanding the scope of case studies to include diverse fields such as renewable energy adoption, transportation emissions, public policy, and financial markets could demonstrate the versatility of the proposed models. Additionally, incorporating real data and advanced computational techniques could facilitate dynamic modeling, allowing for more responsive and adaptive strategies in uncertain environments.

## Figures and Tables

**Table 1:** The equilibrium value of various belief degree β.

β	*k*	$J(0,x_{0})$ Without State Delays and V-Jumps	Equilibrium Value $J(0,x_{0})$
0.25	0.6875	4467.0	15,523.0
0.30	0.6250	2140.9	6493.7
0.35	0.5625	1127.7	2978.5
0.50	0.5000	242.4	499.4
0.75	0.4167	45.2	60.2
0.80	0.3333	35.2	42.5
0.85	0.2500	27.7	31.2

## Data Availability

Data are contained within the article.

## Appendix A

**Lemma A1.** 
*Let $\Upsilon \left(t,x,y\right):\left[0,+\infty \right)\times R^{n}\times R^{k}\to R^{n}$ be a continuously differentiable function, and given the feasible strategy pairs $\omega_{1}\left(t\right)\in \Omega_{1}$ and $\omega_{2}\left(t\right)\in \Omega_{2}$, here, $\left(X_{t},Y_{t}\right)$ follows $dX_{t}=\rho_{1}\left(t,X_{t},Y_{t},\omega_{1},\omega_{2}\right)dt+\rho_{2}\left(X_{t},Y_{t}\right)\varrho_{t}dt+\varsigma (t,X_{t},Y_{t},\omega_{1},$ $\omega_{2})dC_{t}+\nu \left(t,X_{t},Y_{t},\omega_{1},\omega_{2}\right)dV_{t}$. Then, the uncertain process $\Upsilon (t,X_{t},Y_{t})$ satisfies*

(A1)
$$\begin{array}{cc} d\Upsilon (t,X_{t},Y_{t})= & \{\Upsilon_{t}\left(t,X_{t},Y_{t}\right)+\Xi_{x}\Upsilon \left(t,X_{t},Y_{t}\right)\left(\rho_{1}\left(t,X_{t},Y_{t},\omega_{1},\omega_{2}\right)+\rho_{2}\left(X_{t},Y_{t}\right)\varrho_{t}\right)\}dt\\ & +\;\Xi_{x}\Upsilon \left(t,X_{t},Y_{t}\right)\varsigma \left(t,X_{t},Y_{t},\omega_{1},\omega_{2}\right)dC_{t}+\;\Xi_{x}\Upsilon \left(t,X_{t},Y_{t}\right)\nu \left(t,X_{t},Y_{t},\omega_{1},\omega_{2}\right)dV_{t}\\ & +\Xi_{y}\Upsilon \left(t,X_{t},Y_{t}\right)\left(F\left(X_{t}\right)-\;e^{-\lambda d}\varrho_{t}-\lambda Y_{t}\right)dt. \end{array}$$

**Proof.** Given the feasible strategy pairs $\left(\omega_{1},\omega_{2}\right)$, and denoting ${\tilde{M}}_{t}$ by ${\tilde{M}}_{t}=\int_{0}^{t}F\left(X_{s}\right)ds,$ then, the process $Y_{t}$ has the representation

$$\begin{array}{cc} Y_{t}= & \int_{-d}^{0}e^{\lambda s}F\left(X_{t+s}\right)ds=\int_{-d}^{0}e^{\lambda s}d{\tilde{M}}_{t+s}=e^{\lambda s}{\tilde{M}}_{t+s}{|}_{-d}^{0}-\int_{-d}^{0}{\tilde{M}}_{t+s}de^{\lambda s}\\ = & {\tilde{M}}_{t}-e^{-\lambda d}{\tilde{M}}_{t-d}-\int_{-d}^{0}\lambda e^{\lambda s}{\tilde{M}}_{t+s}ds\\ = & \int_{0}^{t}F\left(X_{s}\right)ds-e^{-\lambda d}\int_{0}^{t-d}F\left(X_{s}\right)ds-\lambda \int_{-d}^{0}e^{\lambda s}\int_{0}^{t+s}F\left(X_{r}\right)drds. \end{array}$$

Thus, $dY_{t}=\left(F\left(X_{t}\right)-e^{-\lambda d}F\left(X_{t-d}\right)-\lambda Y_{t}\right)dt=\left(F\left(X_{t}\right)-e^{-\lambda d}\varrho_{t}-\lambda Y_{t}\right)dt.$ Applying Lemma 2 to $\Upsilon (t,X_{t},Y_{t}),$ Equation (A1) follows. Thus, the dynamics for $Z_{t}=Z\left(X_{t},Y_{t}\right)$ by using (9) and (A1) can be expressed as follows.

(A2) $$\begin{array}{c} \begin{array}{cc} dZ_{t} & =dZ(X_{t},Y_{t})\\ & =\Xi_{x}Z\left(X_{t},Y_{t}\right)\left(\rho_{1}\left(t,X_{t},Y_{t},\omega_{1},\omega_{2}\right)+\rho_{2}\left(X_{t},Y_{t}\right)\varrho_{t}\right)dt\\ & \;+\Xi_{x}Z\left(X_{t},Y_{t}\right)\varsigma \left(t,X_{t},Y_{t},\omega_{1},\omega_{2}\right)dC_{t}+\Xi_{x}Z\left(X_{t},Y_{t}\right)\nu \left(t,X_{t},Y_{t},\omega_{1},\omega_{2}\right)dV_{t}\\ & \;+\Xi_{y}Z\left(X_{t},Y_{t}\right)\left(F\left(X_{t}\right)-e^{-\lambda d}\varrho_{t}-\lambda Y_{t}\right)dt\\ & =\Xi_{x}Z\left(X_{t},Y_{t}\right)\rho_{1}\left(t,X_{t},Y_{t},\omega_{1},\omega_{2}\right)dt+\Xi_{y}Z\left(X_{t},Y_{t}\right)\left(F\left(X_{t}\right)-\lambda Y_{t}\right)dt\\ & \;+\Xi_{x}Z\left(X_{t},Y_{t}\right)\varsigma \left(t,X_{t},Y_{t},\omega_{1},\omega_{2}\right)dC_{t}+\Xi_{x}Z\left(X_{t},Y_{t}\right)\nu \left(t,X_{t},Y_{t},\omega_{1},\omega_{2}\right)dV_{t}. \end{array} \end{array}$$

□

**Lemma A2.** 
*Suppose that Assumptions 1 and 2 hold and ${\bar{J}}_{t}\left(t,z\right)$ is twice differentiable on $\left[0,T\right]\times R^{n}.$ Then, we have*

(A3)
$$\begin{array}{c} \left\{\begin{array}{c} -{\bar{J}}_{1t}\left(t,z\right)=\underset{\omega_{1}\left(t\right)\in \Omega_{1}}{\sup}\{{\bar{F}}_{1}\left(t,z,\omega_{1}\left(t\right),\omega_{2}\left(t\right)\right)+\nabla_{z}{\bar{J}}_{1}{\left(t,z\right)}^{\tau }\bar{\rho }\left(t,z,\omega_{1}\left(t\right),\omega_{2}\left(t\right)\right)\\ \;\;\;\;\;\;+\frac{\sqrt{3}}{\pi }\ln\frac{1-\beta }{\beta }∥\nabla_{z}{\bar{J}}_{2}{\left(t,z\right)}^{\tau }\bar{\varsigma }\left(t,z,\omega_{1}\left(t\right),\omega_{2}\left(t\right)\right){∥}_{1}+k∥\nabla_{z}{\bar{J}}_{1}{\left(t,z\right)}^{\tau }\bar{\nu }\left(t,z,\omega_{1}\left(t\right),\omega_{2}\left(t\right)\right)∥\}\\ {\bar{J}}_{1}\left(T,Z_{T}\right)={\bar{h}}_{1}\left(Z_{T}\right), \end{array}\right. \end{array}$$

(A4)
$$\begin{array}{c} \left\{\begin{array}{c} -{\bar{J}}_{2t}\left(t,z\right)=\underset{\omega_{2}\left(t\right)\in \Omega_{2}}{\sup}\{{\bar{F}}_{2}\left(t,z,\omega_{1}\left(t\right),\omega_{2}\left(t\right)\right)+\nabla_{z}{\bar{J}}_{2}{\left(t,z\right)}^{\tau }\bar{\rho }\left(t,z,\omega_{1}\left(t\right),\omega_{2}\left(t\right)\right)\\ \;\;\;\;\;\;+\frac{\sqrt{3}}{\pi }\ln\frac{1-\beta }{\beta }∥\nabla_{z}{\bar{J}}_{2}{\left(t,z\right)}^{\tau }\bar{\varsigma }\left(t,z,\omega_{1}\left(t\right),\omega_{2}\left(t\right)\right){∥}_{1}+k∥\nabla_{z}{\bar{J}}_{2}{\left(t,z\right)}^{\tau }\bar{\nu }\left(t,z,\omega_{1}\left(t\right),\omega_{2}\left(t\right)\right)∥\}\\ {\bar{J}}_{2}\left(T,Z_{T}\right)={\bar{h}}_{2}\left(Z_{T}\right), \end{array}\right. \end{array}$$

*and ${\bar{J}}_{i}\left(t,z\right)=J_{i}\left(t,\varphi_{t}\right),$ where ${\bar{J}}_{it}\left(t,z\right)$ is the partial derivative of the function ${\bar{J}}_{i}\left(t,z\right)$ in t, and $\nabla_{z}{\bar{J}}_{i}\left(t,z\right)$ is the gradient of ${\bar{J}}_{i}\left(t,z\right)$ in z.*

(1) *If $\nabla_{z}{\bar{J}}_{i}^{\tau }\bar{\nu }\geq 0$, then $k=1-\frac{\beta }{2(1-\vartheta_{2})}\left(0<\beta <1-\vartheta_{2}\right)$ or $k=\frac{1}{2}\left(1-\vartheta_{2}\leq \beta <1-\vartheta_{1}\right)$ or $k=\frac{1-\beta }{2\vartheta_{1}}\left(1-\vartheta_{1}\leq \beta <1\right)$;*
(2) *if $\nabla_{z}{\bar{J}}_{i}^{\tau }\bar{\nu }<0$, then $k=\frac{\beta }{2\vartheta_{1}}\left(0<\beta \leq \vartheta_{1}\right)$ or $k=\frac{1}{2}\left(\vartheta_{1}<\beta \leq \vartheta_{2}\right)$ or $k=1-\frac{1-\beta }{2(1-\vartheta_{2})}\left(\vartheta_{2}<\beta <1\right)$.*

**Proof.** By Lemma 1, Equations (A3) and (A4) hold. Furthermore, if $\forall \omega_{1}\left(t\right)\in \Omega_{1}$, $\omega_{2}\left(t\right)\in \Omega_{2}$, it holds that

$$\begin{array}{c} \begin{array}{cc} \bar{J}\left(t,z\right) & \geq {\left\{\int_{t}^{T}\bar{F}\left(s,Z_{s},\omega_{1}\left(s\right),\omega_{2}\left(s\right)\right)ds+\bar{h}\left(Z_{T}\right)\right\}}_{\sup}\left(\beta \right)\\ & ={\left\{\int_{t}^{T}F\left(s,X_{s},Y_{s},\omega_{1}\left(s\right),\omega_{2}\left(s\right)\right)ds+h\left(X_{T},Y_{T}\right)\right\}}_{\sup}\left(\beta \right). \end{array} \end{array}$$

Thus, $\bar{J}\left(t,z\right)\geq \underset{\omega_{i}\in \Omega_{i}}{\sup}{\left\{\int_{t}^{T}F\left(s,X_{s},Y_{s},\omega_{1}\left(s\right),\omega_{2}\left(s\right)\right)ds+h\left(X_{T},Y_{T}\right)\right\}}_{\sup}\left(\beta \right)=J\left(t,\varphi_{t}\right),i=1,2.$ In the same way, we obtain that $J\left(t,\varphi_{t}\right)\geq \bar{J}\left(t,z\right).$ □

**Proof of Theorem** **1.** Let $Z\left(x,y\right)=x+e^{\lambda d}A_{3}y$; then, Assumption 1 holds. Further, we have

(A5) $$\begin{array}{c} \left\{\begin{array}{c} \tilde{\rho }\left(t,x,y,\omega_{1},\omega_{2}\right)=\Xi_{x}Z\left(x,y\right)\rho_{1}\left(t,x,y,\omega_{1},\omega_{2}\right)+\Xi_{y}Z\left(x,y\right)\left(x-\lambda y\right)\\ \;\;\;\;\;\;\;=\left[A_{1}+e^{\lambda d}A_{3}\right]\left[Z\left(x,y\right)-e^{\lambda d}A_{3}y\right]+\left(A_{2}-\lambda e^{\lambda d}\right)y+B_{1}\omega_{1}+B_{2}\omega_{2}\\ \;\;\;\;\;\;\;=\left[A_{1}+e^{\lambda d}A_{3}\right]\left[x+e^{\lambda d}A_{3}y\right]-\left[A_{1}e^{\lambda d}A_{3}-A_{2}+e^{\lambda d}A_{3}\left(e^{\lambda d}A_{3}+\lambda \right)\right]y\\ \;\;\;\;\;\;\;\;+B_{1}\omega_{1}+B_{2}\omega_{2}\\ {\tilde{F}}_{i}\left(t,z,\omega_{1},\omega_{2}\right)=z^{\tau }D_{i}z+\omega_{1}^{\tau }R_{i1}\omega_{1}+\omega_{2}^{\tau }R_{i2}\omega_{2},i=1,2,\\ {\tilde{h}}_{i}\left(z\right)=z_{T}^{\tau }G_{i}z_{T},\\ \;\tilde{\varsigma }\left(t,x,y,\omega_{1},\omega_{2}\right)=\Xi_{x}z\left(x,y\right)\varsigma \left(t,x,y,\omega_{1},\omega_{2}\right)=A_{4}z+\left(A_{5}-A_{4}e^{\lambda d}A_{3}\right)y\\ \;\tilde{\nu }\left(t,x,y,\omega_{1},\omega_{2}\right)=\Xi_{x}z\left(x,y\right)\nu \left(t,x,y,\omega_{1},\omega_{2}\right)=A_{6}z+\left(A_{7}-A_{6}e^{\lambda d}A_{3}\right)y. \end{array}\right. \end{array}$$

Therefore, Assumption 2 holds if and only if:

$$A_{2}\left(t\right)=e^{\lambda d}\left(\lambda I+A_{1}\left(t\right)+e^{\lambda d}A_{3}\left(t\right)\right)A_{3}\left(t\right)$$

$$A_{5}\left(t\right)=e^{\lambda d}A_{4}\left(t\right)A_{3}\left(t\right)$$

$$A_{7}\left(t\right)=e^{\lambda d}A_{6}\left(t\right)A_{3}\left(t\right)$$

Then, the following finite-dimensional Nash equilibrium problem can be obtained by the transformation of $Z\left(x,y\right)=x+e^{\lambda d}A_{3}y$.

(A6) $$\begin{array}{c} \left(\bar{NP}\right)\left[\begin{array}{c} {\bar{J}}_{i}\left(t,z\right)=\underset{\omega_{i}\in \Omega_{i}}{\sup}{\left\{\int_{t}^{T}\left[z^{\tau }D_{i}z+\omega_{1}^{\tau }R_{i1}\omega_{1}+\omega_{2}^{\tau }R_{i2}\omega_{2}\right]ds+z_{T}^{\tau }G_{i}z_{T}\right\}}_{\sup}\left(\beta \right)\\ \;\mathrm{subject}\;\;\mathrm{to}\;\\ \;dZ_{s}=\left\{\left(A_{1}\left(s\right)+e^{\lambda d}A_{3}\right)Z_{s}+B_{1}\omega_{1}+B_{2}\omega_{2}\right\}ds\\ \;\;\;\;+A_{4}\left(s\right)zdC_{s}+A_{6}\left(s\right)zdV_{s},\;s\in \left[t,T\right]\\ \;Z_{t}=z,\\ \;\omega_{1}\left(s\right)\in \Omega_{1},\omega_{2}\left(s\right)\in \Omega_{2},\;s\in \left[t,T\right]. \end{array}\right. \end{array}$$

It follows from the optimality condition of Lemma A2 that:

$$\begin{array}{cc} -{\bar{J}}_{it}\left(t,z\right)= & \sup\{{\bar{F}}_{i}\left(t,z,\omega_{1}\left(t\right),\omega_{2}\left(t\right)\right)+\nabla_{z}{\bar{J}}_{i}{\left(t,z\right)}^{\tau }\bar{\rho }\left(t,z,\omega_{1}\left(t\right),\omega_{2}\left(t\right)\right)\}\\ & +\nabla_{z}{\bar{J}}_{i}{\left(t,z\right)}^{\tau }k\bar{\nu }\left(t,z,,\omega_{1}\left(t\right),\omega_{2}\left(t\right)\right)\}, \end{array}$$

that is,

(A7) $$\begin{array}{cc} -{\bar{J}}_{1t}\left(t,z\right)= & \underset{\omega_{1}\in \Omega_{1}}{\sup}\{z^{\tau }D_{1}z+\omega_{1}^{\tau }R_{11}\omega_{1}+\omega_{2}^{\tau }R_{12}\omega_{2}+\nabla_{z}{\bar{J}}_{1}{\left(t,z\right)}^{\tau }\left[\left(A_{1}\left(s\right)+e^{\lambda d}A_{3}\right)z+B_{1}\omega_{1}+B_{2}\omega_{2}\right]\\ \; & \;\;+\nabla_{z}{\bar{J}}_{1}{\left(t,z\right)}^{\tau }kA_{6}\left(s\right)z\}\\ = & \underset{\omega_{1}\in \Omega_{1}}{\sup}g_{1}\left(z,\omega_{1},\omega_{2}\right), \end{array}$$

(A8) $$\begin{array}{c} -{\bar{J}}_{2t}\left(t,z\right)=\underset{\omega_{2}\in \Omega_{2}}{\sup}g_{2}\left(z,\omega_{1},\omega_{2}\right), \end{array}$$

where

$$\begin{array}{cc} g_{1}\left(z,\omega_{1},\omega_{2}\right)= & z^{\tau }D_{1}z+\omega_{1}^{\tau }R_{11}\omega_{1}+\omega_{2}^{\tau }R_{12}\omega_{2}+\nabla_{z}{\bar{J}}_{1}{\left(t,z\right)}^{\tau }\left[\left(A_{1}\left(s\right)+e^{\lambda d}A_{3}\right)z+B_{1}\omega_{1}+B_{2}\omega_{2}\right]\\ \; & +\frac{\sqrt{3}}{\pi }\ln\frac{1-\beta }{\beta }\left|\nabla_{z}{\bar{J}}_{1}{\left(t,z\right)}^{\tau }A_{4}\left(s\right)z\right|+\nabla_{z}{\bar{J}}_{1}{\left(t,z\right)}^{\tau }kA_{6}\left(s\right)z,\\ g_{1}\left(z,\omega_{1},\omega_{2}\right)= & z^{\tau }D_{2}z+\omega_{1}^{\tau }R_{21}\omega_{1}+\omega_{2}^{\tau }R_{22}\omega_{2}+\nabla_{z}{\bar{J}}_{2}{\left(t,z\right)}^{\tau }\left[\left(A_{1}\left(s\right)+e^{\lambda d}A_{3}\right)z+B_{1}\omega_{1}+B_{2}\omega_{2}\right]\\ \; & +\frac{\sqrt{3}}{\pi }\ln\frac{1-\beta }{\beta }\left|\nabla_{z}{\bar{J}}_{2}{\left(t,z\right)}^{\tau }A_{4}\left(s\right)z\right|+\nabla_{z}{\bar{J}}_{2}{\left(t,z\right)}^{\tau }kA_{6}\left(s\right)z, \end{array}$$

where *k* depends on $A_{6}\left(t\right)$. Owing to $\nabla_{z}J^{\tau }A_{6}\left(t\right)z=A_{6}\left(t\right)$,

(1) if $A_{6}\left(t\right)\geq 0$, then $k=1-\frac{\beta }{2(1-\vartheta_{2})}\left(0<\beta <1-\vartheta_{2}\right)$ or $k=\frac{1}{2}\left(1-\vartheta_{2}\leq \beta <1-\vartheta_{1}\right)$ or $k=\frac{1-\beta }{2\vartheta_{1}}\left(1-\vartheta_{1}\leq \beta <1\right)$;
(2) if $A_{6}\left(t\right)<0$, then $k=\frac{\beta }{2\vartheta_{1}}\left(0<\beta \leq \vartheta_{1}\right)$ or $k=\frac{1}{2}\left(\vartheta_{1}<\beta \leq \vartheta_{2}\right)$ or $k=1-\frac{1-\beta }{2(1-\vartheta_{2})}\left(\vartheta_{2}<\beta <1\right)$. Let

(A9) $$\begin{array}{c} \left\{\begin{array}{c} \frac{\partial g_{1}\left(z,\omega_{1},\omega_{2}\right)}{\partial \omega_{1}}=2R_{11}\omega_{1}+B_{1}^{\tau }\nabla_{z}{\bar{J}}_{1}=0\\ \frac{\partial g_{2}\left(z,\omega_{1},\omega_{2}\right)}{\partial \omega_{2}}=2R_{22}\omega_{2}+B_{2}^{\tau }\nabla_{z}{\bar{J}}_{2}=0, \end{array}\right. \end{array}$$

we can obtain that

(A10) $$\begin{array}{c} \left\{\begin{array}{c} \omega_{1}^{*}\left(t\right)=-\frac{1}{2}R_{11}^{-1}B_{1}^{\tau }\nabla_{z}{\bar{J}}_{1}\\ \omega_{2}^{*}\left(t\right)=-\frac{1}{2}R_{22}^{-1}B_{2}^{\tau }\nabla_{z}{\bar{J}}_{2}. \end{array}\right. \end{array}$$

Owing to ${\bar{J}}_{i}\left(T,z_{T}\right)=z_{T}^{\tau }G_{i}z_{T},i=1,2$, we can guess

$${\bar{J}}_{i}\left(t,z\right)=z^{\tau }Q_{i}\left(t\right)z,$$

Then,

$$\nabla_{z}{\bar{J}}_{i}\left(t,z\right)=2Q_{i}\left(t\right)z.$$

Thus, (A10) can be written as:

(A11) $$\begin{array}{c} \left\{\begin{array}{c} \omega_{1}^{*}\left(t\right)=-R_{11}^{-1}B_{1}^{\tau }Q_{1}\left(t\right)z\\ \omega_{2}^{*}\left(t\right)=-R_{22}^{-1}B_{2}^{\tau }Q_{2}\left(t\right)z. \end{array}\right. \end{array}$$

On the other hand, there is:

(A12) $$\begin{array}{c} {\bar{J}}_{it}=z^{\tau }{\dot{Q}}_{i}\left(t\right)z. \end{array}$$

We denote

$$\begin{array}{cc} \Delta_{1}= & \left\{\left(t,z\right)|z^{\tau }Q_{i}\left(t\right)A_{4}\left(t\right)z\geq 0,\left(t,z\right)\in \left[0,T\right]\times {\left[a,b\right]}^{n}\right\},\\ \Delta_{2}= & \left\{\left(t,z\right)|z^{\tau }Q_{i}\left(t\right)A_{4}\left(t\right)z<0,\left(t,z\right)\in \left[0,T\right]\times {\left[a,b\right]}^{n}\right\}. \end{array}$$

If $\left(t,z\right)\in \Delta_{1}$, we substitute (A11) and (A12) into (A7) and (A8), and it holds that

$$\begin{array}{cc} -z^{\tau }{\dot{Q}}_{1}\left(t\right)z= & z^{\tau }\{D_{1}+Q_{1}^{\tau }B_{1}R_{11}^{-1}B_{1}^{\tau }Q_{1}+Q_{2}^{\tau }B_{2}R_{22}^{-1}R_{12}R_{22}^{-1}B_{2}^{\tau }Q_{2}\\ \; & +2Q_{1}^{\tau }\left(A_{1}+e^{\lambda d}A_{3}+\frac{\sqrt{3}}{\pi }\ln\frac{1-\beta }{\beta }A_{4}+kA_{6}\right)-2Q_{1}^{\tau }B_{1}R_{11}^{-1}B_{1}^{\tau }Q_{1}-2Q_{1}^{\tau }B_{2}R_{22}^{-1}B_{2}^{\tau }Q_{2}\}z, \end{array}$$

$$\begin{array}{cc} -z^{\tau }{\dot{Q}}_{2}\left(t\right)z= & z^{\tau }\{D_{2}+Q_{2}^{\tau }B_{2}R_{22}^{-1}B_{2}^{\tau }Q_{2}+Q_{1}^{\tau }B_{1}R_{11}^{-1}R_{21}R_{11}^{-1}B_{1}^{\tau }Q_{1}\\ \; & +2Q_{2}^{\tau }\left(A_{1}+e^{\lambda d}A_{3}+\frac{\sqrt{3}}{\pi }\ln\frac{1-\beta }{\beta }A_{4}+kA_{6}\right)-2Q_{2}^{\tau }B_{1}R_{11}^{-1}B_{1}^{\tau }Q_{1}-2Q_{2}^{\tau }B_{2}R_{22}^{-1}B_{2}^{\tau }Q_{2}\}z. \end{array}$$

Considering that ${\bar{J}}_{i}\left(T,z_{T}\right)=z_{T}^{\tau }G_{i}z_{T}$, we obtain

$$\begin{array}{c} \left\{\begin{array}{c} {\dot{Q}}_{1}\left(t\right)=-\{D_{1}-Q_{1}^{\tau }B_{1}R_{11}^{-1}B_{1}^{\tau }Q_{1}+Q_{2}^{\tau }B_{2}R_{22}^{-1}R_{12}R_{22}^{-1}B_{2}^{\tau }Q_{2}+Q_{1}^{\tau }(A_{1}+e^{\lambda d}A_{3}+\frac{\sqrt{3}}{\pi }\ln\frac{1-\beta }{\beta }A_{4}\\ \;\;\;\;+kA_{6})+{\left(A_{1}+e^{\lambda d}A_{3}+\frac{\sqrt{3}}{\pi }\ln\frac{1-\beta }{\beta }A_{4}+kA_{6}\right)}^{\tau }Q_{1}-2Q_{1}^{\tau }B_{2}R_{22}^{-1}B_{2}^{\tau }Q_{2}\},\\ Q_{1}\left(T\right)=G_{1},\\ {\dot{Q}}_{2}\left(t\right)=-\{D_{2}-Q_{2}^{\tau }B_{2}R_{22}^{-1}B_{2}^{\tau }Q_{2}+Q_{1}^{\tau }B_{1}R_{11}^{-1}R_{21}R_{11}^{-1}B_{1}^{\tau }Q_{1}+Q_{2}^{\tau }(A_{1}+e^{\lambda d}A_{3}+\frac{\sqrt{3}}{\pi }\ln\frac{1-\beta }{\beta }A_{4}\\ \;\;\;\;+kA_{6})+{\left(A_{1}+e^{\lambda d}A_{3}+\frac{\sqrt{3}}{\pi }\ln\frac{1-\beta }{\beta }A_{4}+kA_{6}\right)}^{\tau }Q_{2}-2Q_{2}^{\tau }B_{1}R_{11}^{-1}B_{1}^{\tau }Q_{1}\},\\ Q_{2}\left(T\right)=G_{2}. \end{array}\right. \end{array}$$

If $\left(t,z\right)\in \Delta_{2}$, we substitute (A11) and (A12) into (A7) and (A8), and it holds that

$$\begin{array}{cc} -z^{\tau }{\dot{Q}}_{1}\left(t\right)z= & z^{\tau }\{D_{1}+Q_{1}^{\tau }B_{1}R_{11}^{-1}B_{1}^{\tau }Q_{1}+Q_{2}^{\tau }B_{2}R_{22}^{-1}R_{12}R_{22}^{-1}B_{2}^{\tau }Q_{2}\\ \; & +2Q_{1}^{\tau }\left(A_{1}+e^{\lambda d}A_{3}-\frac{\sqrt{3}}{\pi }\ln\frac{1-\beta }{\beta }A_{4}+kA_{6}\right)-2Q_{1}^{\tau }B_{1}R_{11}^{-1}B_{1}^{\tau }Q_{1}-2Q_{1}^{\tau }B_{2}R_{22}^{-1}B_{2}^{\tau }Q_{2}\}z, \end{array}$$

$$\begin{array}{cc} -z^{\tau }{\dot{Q}}_{2}\left(t\right)z= & z^{\tau }\{D_{2}+Q_{2}^{\tau }B_{2}R_{22}^{-1}B_{2}^{\tau }Q_{2}+Q_{1}^{\tau }B_{1}R_{11}^{-1}R_{21}R_{11}^{-1}B_{1}^{\tau }Q_{1}\\ \; & +2Q_{2}^{\tau }\left(A_{1}+e^{\lambda d}A_{3}-\frac{\sqrt{3}}{\pi }\ln\frac{1-\beta }{\beta }A_{4}+kA_{6}\right)-2Q_{2}^{\tau }B_{1}R_{11}^{-1}B_{1}^{\tau }Q_{1}-2Q_{2}^{\tau }B_{2}R_{22}^{-1}B_{2}^{\tau }Q_{2}\}z. \end{array}$$

Considering that ${\bar{J}}_{i}\left(T,z_{T}\right)=z_{T}^{\tau }G_{i}z_{T}$, we obtain

$$\begin{array}{c} \left\{\begin{array}{c} {\dot{Q}}_{1}\left(t\right)=-\{D_{1}-Q_{1}^{\tau }B_{1}R_{11}^{-1}B_{1}^{\tau }Q_{1}+Q_{2}^{\tau }B_{2}R_{22}^{-1}R_{12}R_{22}^{-1}B_{2}^{\tau }Q_{2}+Q_{1}^{\tau }(A_{1}+e^{\lambda d}A_{3}-\frac{\sqrt{3}}{\pi }\ln\frac{1-\beta }{\beta }A_{4}\\ \;\;\;\;+kA_{6})+{\left(A_{1}+e^{\lambda d}A_{3}-\frac{\sqrt{3}}{\pi }\ln\frac{1-\beta }{\beta }A_{4}+kA_{6}\right)}^{\tau }Q_{1}-2Q_{1}^{\tau }B_{2}R_{22}^{-1}B_{2}^{\tau }Q_{2}\},\\ Q_{1}\left(T\right)=G_{1},\\ {\dot{Q}}_{2}\left(t\right)=-\{D_{2}-Q_{2}^{\tau }B_{2}R_{22}^{-1}B_{2}^{\tau }Q_{2}+Q_{1}^{\tau }B_{1}R_{11}^{-1}R_{21}R_{11}^{-1}B_{1}^{\tau }Q_{1}+Q_{2}^{\tau }(A_{1}+e^{\lambda d}A_{3}-\frac{\sqrt{3}}{\pi }\ln\frac{1-\beta }{\beta }A_{4}\\ \;\;\;\;+kA_{6})+{\left(A_{1}+e^{\lambda d}A_{3}-\frac{\sqrt{3}}{\pi }\ln\frac{1-\beta }{\beta }A_{4}+kA_{6}\right)}^{\tau }Q_{2}-2Q_{2}^{\tau }B_{1}R_{11}^{-1}B_{1}^{\tau }Q_{1}\},\\ Q_{2}\left(T\right)=G_{2}. \end{array}\right. \end{array}$$

The proof is completed. □

For problem (27)–(29), we first give the equilibrium equation of the optimistic value model as follows:

**Lemma A3.** 
*Suppose that Assumptions 1 and 3 hold and $\bar{J}\left(t,z\right)$ is twice differentiable on [0,T]×R. Then, the equilibrium equation holds:*

(A13)
$$\begin{array}{cc} -{\bar{J}}_{t}\left(t,z\right) & =\max_{\omega_{1}\in \Omega_{1}}\min_{\omega_{2}\in \Omega_{2}}\left\{{\bar{J}}_{z}{\left(t,z\right)}^{\tau }\bar{\rho }\left(t,z,\omega_{1},\omega_{2}\right)+\bar{F}\left(t,z,\omega_{1},\omega_{2}\right)\right.\\ & +\frac{\sqrt{3}}{\pi }\ln\frac{1-\beta }{\beta }\left|J_{z}{\left(t,z\right)}^{\tau }\bar{\varsigma }\left(t,z,\omega_{1},\omega_{2}\right)\right|+k{\bar{J}}_{z}{\left(t,z\right)}^{\tau }\bar{\nu }\left(t,z,\omega_{1},\omega_{2}\right)\} \end{array}$$

(A14)
$$\begin{array}{c} =\min_{\omega_{2}\in \Omega_{2}}\max_{\omega_{1}\in \Omega_{1}}\left\{{\bar{J}}_{z}{\left(t,z\right)}^{\tau }\bar{\rho }\left(t,z,\omega_{1},\omega_{2}\right)+\bar{F}\left(t,z,\omega_{1},\omega_{2}\right)\right.\\ +\frac{\sqrt{3}}{\pi }\ln\frac{1-\beta }{\beta }\left|{\bar{J}}_{z}{\left(t,z\right)}^{\tau }\bar{\varsigma }\left(t,z,\omega_{1},\omega_{2}\right)\right|+k{\bar{J}}_{z}{\left(t,z\right)}^{\tau }\bar{\nu }\left(t,z,\omega_{1},\omega_{2}\right)\}. \end{array}$$

**Proof.** Assume that $\left(\omega_{1}^{*},\omega_{2}^{*}\right)$ are the strategy pairs for (27)–(29). In addition, $\omega_{1}^{*}$ and $\omega_{2}^{*}$ satisfy (P1) and (P2):

$$\begin{array}{c} \left(P1\right)\;\left\{\begin{array}{c} \bar{J}\left(t,z\right)\equiv \max_{\omega_{1}\in \Omega_{1}}\hat{J}\left(\omega_{1},\omega_{2}^{*}\right)\\ \;\mathrm{subject}\;\;\mathrm{to}:\;\\ \;dz_{s}=\bar{\rho }\left(s,z_{s},\omega_{1},\omega_{2}^{*}\right)ds+\bar{\varsigma }\left(s,z_{s},\omega_{1},\omega_{2}^{*}\right)dC_{s}+\bar{\nu }\left(s,z_{s},\omega_{1},\omega_{2}^{*}\right)dV_{s}\;\;\mathrm{and}\;\;z_{t}=z, \end{array}\right. \end{array}$$

and

$$\begin{array}{c} \left(P2\right)\;\left\{\begin{array}{c} \bar{J}\left(t,z\right)\equiv \min_{\omega_{2}\in \Omega_{2}}\hat{J}\left(\omega_{1}^{*},\omega_{2}\right)\\ \;\mathrm{subject}\;\;\mathrm{to}:\;\\ \;dz_{s}=\bar{\rho }\left(s,z_{s},\omega_{1}^{*},\omega_{2}\right)ds+\bar{\varsigma }\left(s,z_{s},\omega_{1}^{*},\omega_{2}\right)dC_{s}+\bar{\nu }\left(s,z_{s},\omega_{1}^{*},\omega_{2}\right)dV_{s}\;\;\mathrm{and}\;\;z_{t}=z. \end{array}\right. \end{array}$$

Applying Lemma 1 to (P1) and (P2), it holds that

(A15) $$\begin{array}{cc} -{\bar{J}}_{t}\left(t,z\right)= & \max_{\omega_{1}\in \Omega_{1}}\{{\bar{J}}_{z}{\left(t,z\right)}^{\tau }\bar{\rho }\left(t,z,\omega_{1},\omega_{2}^{*}\right)+\bar{F}\left(t,z,\omega_{1},\omega_{2}^{*}\right)\\ & +\frac{\sqrt{3}}{\pi }\ln\frac{1-\beta }{\beta }\left|{\bar{J}}_{z}{\left(t,z\right)}^{\tau }\bar{\varsigma }\left(t,z,\omega_{1},\omega_{2}^{*}\right)\right|+k{\bar{J}}_{z}{\left(t,x\right)}^{\tau }\bar{\nu }\left(t,z,\omega_{1},\omega_{2}^{*}\right)\} \end{array}$$

(A16) $$\begin{array}{cc} = & \min_{\omega_{2}\in \Omega_{2}}\{{\bar{J}}_{z}{\left(t,z\right)}^{\tau }\bar{\rho }\left(t,z,\omega_{1}^{*},\omega_{2}\right)+\bar{F}\left(t,z,\omega_{1}^{*},\omega_{2}\right)\\ & +\frac{\sqrt{3}}{\pi }\ln\frac{1-\beta }{\beta }\left|{\bar{J}}_{z}{\left(t,z\right)}^{\tau }\bar{\varsigma }\left(t,z,\omega_{1}^{*},\omega_{2}\right)\right|+k{\bar{J}}_{z}{\left(t,z\right)}^{\tau }\bar{\nu }\left(t,z,\omega_{1}^{*},\omega_{2}\right)\}. \end{array}$$

By (A15), it holds that

(A17) $$\begin{array}{cc} -{\bar{J}}_{t}\left(t,z\right)\geq & \max_{\omega_{1}\in \Omega_{1}}\min_{\omega_{2}\in \Omega_{2}}\{{\bar{J}}_{z}{\left(t,z\right)}^{\tau }\bar{\rho }\left(t,z,\omega_{1},\omega_{2}\right)+\bar{F}\left(t,z,\omega_{1},\omega_{2}\right)\\ & \;+\frac{\sqrt{3}}{\pi }\ln\frac{1-\beta }{\beta }\left|{\bar{J}}_{z}{\left(t,z\right)}^{\tau }\bar{\varsigma }\left(t,z,\omega_{1},\omega_{2}\right)\right|+k{\bar{J}}_{z}{\left(t,z\right)}^{\tau }\bar{\nu }\left(t,z,\omega_{1},\omega_{2}\right)\}. \end{array}$$

Similarly, from (A16), it holds that

(A18) $$\begin{array}{cc} -{\bar{J}}_{t}\left(t,z\right)\leq & \min_{\omega_{2}\in \Omega_{2}}\max_{\omega_{1}\in \Omega_{1}}\{{\bar{J}}_{z}{\left(t,z\right)}^{\tau }\bar{\rho }\left(t,z,\omega_{1},\omega_{2}\right)+\bar{F}\left(t,z,\omega_{1},\omega_{2}\right)\\ & \;\;+\frac{\sqrt{3}}{\pi }\ln\frac{1-\beta }{\beta }\left|{\bar{J}}_{z}{\left(t,z\right)}^{\tau }\bar{\varsigma }\left(t,z,\omega_{1},\omega_{2}\right)\right|+k{\bar{J}}_{z}{\left(t,z\right)}^{\tau }\bar{\nu }\left(t,z,\omega_{1},\omega_{2}\right)\}. \end{array}$$

Let

$$\begin{array}{cc} L(\omega_{1},\omega_{2})= & {\bar{J}}_{z}{\left(t,z\right)}^{\tau }\bar{\rho }\left(t,z,\omega_{1},\omega_{2}\right)+\bar{F}\left(t,z,\omega_{1},\omega_{2}\right)\\ & +\frac{\sqrt{3}}{\pi }\ln\frac{1-\beta }{\beta }\left|{\bar{J}}_{z}{\left(t,z\right)}^{\tau }\bar{\varsigma }\left(t,z,\omega_{1},\omega_{2}\right)\right|+k{\bar{J}}_{z}{\left(t,z\right)}^{\tau }\bar{\nu }\left(t,z,\omega_{1},\omega_{2}\right). \end{array}$$

We note that

(A19) $$\begin{array}{c} \max_{\omega_{1}\in \Omega_{1}}\min_{\omega_{2}\in \Omega_{2}}L\left(\omega_{1},\omega_{2}\right)\geq \min_{\omega_{2}\in \Omega_{2}}L\left(\omega_{1},\omega_{2}\right),\;\forall \omega_{1}. \end{array}$$

By (A19), we obtain

$$\begin{array}{c} \max_{\omega_{1}\in \Omega_{1}}\min_{\omega_{2}\in \Omega_{2}}L\left(\omega_{1},\omega_{2}\right)\geq \min_{\omega_{2}\in \Omega_{2}}\max_{\omega_{1}\in \Omega_{1}}L\left(\omega_{1},\omega_{2}\right). \end{array}$$

Together with (A17) and (A18), the lemma is completed. □

**Proof of Theorem** **3.** First, we verify the necessity. Let $Z\left(x,y\right)=x+e^{\lambda d}A_{3}y$, then Assumption 1 holds. Further, we have

(A20) $$\begin{array}{c} \left\{\begin{array}{c} \;\tilde{\rho }\left(t,x,y,\omega_{1},\omega_{2}\right)=Z_{x}\left(x,y\right)\rho_{1}\left(t,x,y,\omega_{1},\omega_{2}\right)+Z_{y}\left(x,y\right)\left(x-\lambda y\right)\\ \;\;\;\;\;\;\;\;=\left[A_{1}\left(t\right)+e^{\lambda d}A_{3}\right]\left[Z\left(x,y\right)-e^{\lambda d}A_{3}y\right]+\left(A_{2}-\lambda e^{\lambda d}\right)y+B_{1}\omega_{1}+B_{2}\omega_{2}\\ \;\;\;\;\;\;\;=\left[A_{1}\left(t\right)+e^{\lambda d}A_{3}\right]\left[x+e^{\lambda d}A_{3}y\right]-\left[A_{1}e^{\lambda d}A_{3}-A_{2}+e^{\lambda d}A_{3}\left(e^{\lambda d}A_{3}+\lambda \right)\right]y\\ \;\;\;\;\;\;\;\;+B_{1}\omega_{1}+B_{2}\omega_{2}\\ \;\tilde{F}\left(t,z,\omega_{1},\omega_{2}\right)=Pz^{2}+G\omega_{2}^{2}-H\omega_{1}^{2},\\ \;\tilde{h}\left(z\right)=\frac{1}{2}\Psi_{f}z^{2},\\ \;\tilde{\varsigma }\left(t,x,y,\omega_{1},\omega_{2}\right)=z_{x}\left(x,y\right)\varsigma \left(t,x,y,\omega_{1},\omega_{2}\right)=A_{4}z+\left(A_{5}-A_{4}e^{\lambda d}A_{3}\right)y\\ \;\tilde{\nu }\left(t,x,y,\omega_{1},\omega_{2}\right)=z_{x}\left(x,y\right)\nu \left(t,x,y,\omega_{1},\omega_{2}\right)=A_{6}z+\left(A_{7}-A_{6}e^{\lambda d}A_{3}\right)y. \end{array}\right. \end{array}$$

Therefore, Assumption 3 holds if and only if $A_{2}\left(t\right)=e^{\lambda d}\left(\lambda +A_{1}\left(t\right)+e^{\lambda d}A_{3}\right)A_{3}$ and $A_{5}\left(t\right)=e^{\lambda d}A_{4}\left(t\right)A_{3}$, $A_{7}\left(t\right)=e^{\lambda d}A_{6}\left(t\right)A_{3}$ hold. Then, the following finite-dimensional saddle equilibrium problem $\left(\bar{NP}\right)$ can be obtained by the transformation of $Z\left(x,y\right)=x+e^{\lambda d}A_{3}y$.

(A21) $$\begin{array}{c} \left(\bar{NP}\right)\left[\begin{array}{c} \bar{J}\left(\omega_{1},\omega_{2}\right)={\left\{\frac{1}{2}\int_{0}^{T}\left(P\left(s\right)Z^{2}+G\left(s\right)\omega_{2}^{2}-H\left(s\right)\omega_{1}^{2}\right)ds+\frac{1}{2}\Psi_{f}Z_{T}^{2}\right\}}_{\sup}\left(\beta \right)\\ \;\mathrm{subject}\;\;\mathrm{to}\;\\ \;dZ_{s}=\left\{\left(A_{1}\left(s\right)+e^{\lambda d}A_{3}\right)Z_{s}+B_{1}\left(s\right)\omega_{1}+B_{2}\left(s\right)\omega_{2}\right\}ds\\ \;\;\;\;+A_{4}\left(s\right)zdC_{s}+A_{6}\left(s\right)zdV_{s},\;s\in \left[t,T\right]\\ \;Z_{t}=z,\\ \;\omega_{1}\left(s\right)\in \Omega_{1},\omega_{2}\left(s\right)\in \Omega_{2},\;s\in \left[t,T\right]. \end{array}\right. \end{array}$$

Since $\bar{J}\left(T,Z_{T}\right)=\frac{1}{2}\Psi_{f}Z_{T}^{2}$ is quadratic about $Z_{T}.$ We guess

$$\begin{array}{c} \bar{J}\left(t,z\right)=\frac{1}{2}Q\left(t\right)z^{2} \end{array}$$

and

$$\begin{array}{c} Q\left(t\right)=\Psi_{f}. \end{array}$$

Applying the equilibrium Equations (A13) and (A14) of Lemma A3, we have

$$\begin{array}{cc} -\frac{1}{2}Q^{\prime }\left(t\right)z^{2}= & \max_{\omega_{1}}\min_{\omega_{2}}\{Q\left(t\right)z\left[\left(A_{1}\left(t\right)+e^{\lambda d}A_{3}\right)z+B_{1}\left(t\right)\omega_{1}+B_{2}\left(t\right)\omega_{2}\right]+\frac{1}{2}P\left(t\right)z^{2}+\frac{1}{2}G\left(t\right)u_{2}^{2}\\ & -\frac{1}{2}H\left(t\right)\omega_{1}^{2}+\frac{\sqrt{3}}{\pi }\ln\frac{1-\beta }{\beta }|Q\left(t\right)z\cdot A_{4}\left(t\right)z\left|+kQ\left(t\right)z\cdot A_{6}\left(t\right)z\right\}. \end{array}$$

By the property of optimistic value and the assumptions P(s)≥0, $A_{4}\left(s\right)>0$, G(s)>0, H(s)>0, it is easy to see Q(t)≥0. Thus,

(A22) $$\begin{array}{cc} -\frac{1}{2}Q^{\prime }\left(t\right)z^{2}= & Q\left(t\right)\left(A_{1}\left(t\right)+e^{\lambda d}A_{3}\right)z^{2}+\frac{1}{2}P\left(t\right)z^{2}+\frac{\sqrt{3}}{\pi }\ln\frac{1-\beta }{\beta }Q\left(t\right)A_{4}\left(t\right)z^{2}+kQ\left(t\right)A_{6}\left(t\right)z^{2}\\ \; & +\max_{\omega_{1}}\left[Q\left(t\right)z\cdot B_{1}\left(t\right)\omega_{1}-\frac{1}{2}H\left(t\right)\omega_{1}^{2}\right]+\min_{\omega_{2}}\left[Q\left(t\right)z\cdot B_{2}\left(t\right)\omega_{2}+\frac{1}{2}G\left(t\right)\omega_{2}^{2}\right]\\ = & \chi_{1}+\max_{\omega_{1}}\chi_{2}+\min_{\omega_{2}}\chi_{3}. \end{array}$$

Since $\chi_{2}$ is strictly concave about $\omega_{1}$, $\chi_{3}$ is strictly convex about $\omega_{2}$. Thus, the equilibrium controls $\omega_{1}$ and $\omega_{2}$ satisfy

$$\begin{array}{c} \frac{\partial \chi_{2}}{\partial \omega_{1}}=Q\left(t\right)z\cdot B_{1}\left(t\right)-H\left(t\right)\omega_{1}=0, \end{array}$$

$$\begin{array}{c} \frac{\partial \chi_{3}}{\partial \omega_{2}}=Q\left(t\right)z\cdot B_{2}\left(t\right)+G\left(t\right)\omega_{2}=0. \end{array}$$

Thus, we obtain the strategy pairs

(A23) $$\begin{array}{c} \omega_{1}^{*}=\frac{Q\left(t\right)B_{1}\left(t\right)}{H(t)}z,\omega_{2}^{*}=-\frac{Q\left(t\right)B_{2}\left(t\right)}{G(t)}z. \end{array}$$

Substituting (A23) into (A22), we obtain

$$\begin{array}{cc} -\frac{1}{2}Q^{\prime }\left(t\right)z^{2}= & -\frac{1}{2}z^{2}(-2Q\left(t\right)\left(A_{1}\left(t\right)+e^{\lambda d}A_{3}\right)-P\left(t\right)-\frac{2\sqrt{3}}{\pi }\ln\frac{1-\beta }{\beta }QA_{4}\left(t\right)\\ & -2kQ\left(t\right)A_{6}\left(t\right)-\frac{Q^{2}\left(t\right)B_{1}^{2}\left(t\right)}{H(t)}+\frac{Q^{2}\left(t\right)B_{2}^{2}\left(t\right)}{G(t)}). \end{array}$$

Thus, it holds that

(A24) $$\begin{array}{c} \left\{\begin{array}{c} \frac{dQ(t)}{dt}+2Q\left(t\right)\left(A_{1}\left(t\right)+e^{\lambda d}A_{3}\right)+P+\frac{Q^{2}\left(t\right)B_{1}^{2}\left(t\right)}{H(t)}-\frac{Q^{2}\left(t\right)B_{2}^{2}\left(t\right)}{G(t)}\\ \;\;\;\;\;\;\;+\frac{2\sqrt{3}}{\pi }\ln\frac{1-\beta }{\beta }Q\left(t\right)A_{4}\left(t\right)+2kA_{6}\left(t\right)Q\left(t\right)=0,\\ Q\left(T\right)=\Psi_{f}, \end{array}\right. \end{array}$$

Owing to ${\bar{J}}_{z}A_{6}\left(t\right)z=A_{6}\left(t\right)$, and $A_{6}\left(t\right)\geq 0$, then $k=1-\frac{\beta }{2(1-\vartheta_{2})}\left(0<\beta <1-\vartheta_{2}\right)$ or $k=\frac{1}{2}\left(1-\vartheta_{2}\leq \beta <1-\vartheta_{1}\right)$ or $k=\frac{1-\beta }{2\vartheta_{1}}\left(1-\vartheta_{1}\leq \beta <1\right)$. Next, we verify the sufficient condition. We let Q(t) satisfy Equation (A24). Then, it holds that

$$\begin{array}{cc} d(QZ_{s}^{2})= & Z_{s}^{2}\dot{Q}ds+2QZ_{s}dZ_{s}\\ = & Z_{s}^{2}\dot{Q}ds+2QZ_{s}\left(\left(A_{1}\left(s\right)+e^{\lambda d}A_{3}\right)Z_{s}+B_{1}\omega_{1}+B_{2}\omega_{2}\right)ds+2QA_{4}Z_{s}^{2}dC_{s}+2QA_{6}Z_{s}^{2}dV_{s}\\ = & Z_{s}^{2}\left(\dot{Q}+2Q\left(A_{1}\left(s\right)+e^{\lambda d}A_{3}\right)\right)ds+2QZ_{s}\left(B_{1}\omega_{1}+B_{2}\omega_{2}\right)ds+2QA_{4}Z_{s}^{2}dC_{s}+2QA_{6}Z_{s}^{2}dV_{s}\\ = & Z_{s}^{2}(-P-\frac{Q^{2}B_{1}^{2}}{H}+\frac{Q^{2}B_{2}^{2}}{G}-\frac{2\sqrt{3}}{\pi }\ln\frac{1-\beta }{\beta }Q|A_{4}\left|-2kA_{6}Q\right)ds\\ & +2QZ_{s}\left(B_{1}\omega_{1}+B_{2}\omega_{2}\right)ds+2QA_{4}Z_{s}^{2}dC_{s}+2QA_{6}Z_{s}^{2}dV_{s}. \end{array}$$

From 0 to *T*, integrating the above equation leads to

$$\begin{array}{cc} \frac{1}{2}\Psi_{f}Z_{T}^{2}= & \frac{1}{2}Q\left(0\right)z_{0}^{2}+\frac{1}{2}\int_{0}^{T}Z_{s}^{2}(-P-\frac{Q^{2}B_{1}^{2}}{H}+\frac{Q^{2}B_{2}^{2}}{G}-\frac{2\sqrt{3}}{\pi }\ln\frac{1-\beta }{\beta }Q|A_{4}\left|-2kA_{6}Q\right)ds\\ \; & +\int_{0}^{T}QZ_{s}\left(B_{1}\omega_{1}+B_{2}\omega_{2}\right)ds+\int_{0}^{T}QA_{4}Z_{s}^{2}dC_{s}+\int_{0}^{T}QA_{6}Z_{s}^{2}dV_{s} \end{array}$$

Substituting it into $\bar{J}\left(\omega_{1},\omega_{2}\right)$ of (A21), we obtain

$$\begin{array}{cc} \bar{J}\left(\omega_{1},\omega_{2}\right)= & [\frac{1}{2}\int_{0}^{T}\left(PZ_{s}^{2}+G\omega_{2}^{2}-H\omega_{1}^{2}\right)ds+\frac{1}{2}Q\left(0\right)z_{0}^{2}\\ \; & +\frac{1}{2}\int_{0}^{T}Z_{s}^{2}\left(-P-\frac{Q^{2}B_{1}^{2}}{H}+\frac{Q^{2}B_{2}^{2}}{G}-\frac{2\sqrt{3}}{\pi }\ln\frac{1-\beta }{\beta }QA_{4}-2kA_{6}Q\right)ds\\ \; & +\int_{0}^{T}QZ_{s}\left(B_{1}\omega_{1}+B_{2}\omega_{2}\right)ds+\int_{0}^{T}QA_{4}Z_{s}^{2}dC_{s}+\int_{0}^{T}QA_{6}Z_{s}^{2}dV_{s}{]}_{\sup}\left(\beta \right)\\ = & [\frac{1}{2}\int_{0}^{T}\left[G{\left(\omega_{2}+\frac{QB_{2}Z_{s}}{G}\right)}^{2}-H{\left(\omega_{1}-\frac{QB_{1}Z_{s}}{H}\right)}^{2}\right]ds+\frac{1}{2}Q\left(0\right)z_{0}^{2}+\int_{0}^{T}QA_{4}Z_{s}^{2}dC_{s}\\ & +\int_{0}^{T}QA_{6}Z_{s}^{2}dV_{s}-\frac{\sqrt{3}}{\pi }\ln\frac{1-\beta }{\beta }\int_{0}^{T}QA_{4}Z_{s}^{2}ds-k\int_{0}^{T}QA_{6}Z_{s}^{2}{ds]}_{\sup}\left(\beta \right). \end{array}$$

Obviously, the equilibrium solutions are $\left(\omega_{1}^{*},\omega_{2}^{*}\right)$ of (A23). Then, the theorem is completed. □
